# Lysine l-lactylation is the dominant lactylation isomer induced by glycolysis

**DOI:** 10.1038/s41589-024-01680-8

**Published:** 2024-07-19

**Authors:** Di Zhang, Jinjun Gao, Zhijun Zhu, Qianying Mao, Zhiqiang Xu, Pankaj K. Singh, Cornelius C. Rimayi, Carlos Moreno-Yruela, Shuling Xu, Gongyu Li, Yi-Cheng Sin, Yue Chen, Christian A. Olsen, Nathaniel W. Snyder, Lunzhi Dai, Lingjun Li, Yingming Zhao

**Affiliations:** 1 State Key Laboratory of Protein and Plant Gene Research, School of Life Sciences, Peking University, Beijing, China; 2 Peking-Tsinghua Center for Life Sciences, Academy for Advanced Interdisciplinary Studies, Peking University, Beijing, China; 3 Ben May Department for Cancer Research, The University of Chicago, Chicago, IL USA; 4 Department of Chemistry, University of Wisconsin-Madison, Madison, WI USA; 5 National Clinical Research Center for Geriatrics and General Practice Ward/International Medical Center Ward, General Practice Medical Center, State Key Laboratory of Biotherapy, West China Hospital, Sichuan University, Chengdu, China; 6 Lewis Katz School of Medicine at Temple University, Department of Cardiovascular Sciences, Center for Metabolic Disease Research, Philadelphia, PA USA; 7 Center for Biopharmaceuticals and Department of Drug Design and Pharmacology, Faculty of Health and Medical Sciences, University of Copenhagen, Copenhagen, Denmark; 8 School of Pharmacy, University of Wisconsin-Madison, Madison, WI USA; 9 Department of Biochemistry, Molecular Biology and Biophysics, The University of Minnesota at Twin Cities, Minneapolis, MN USA; 10 Present Address: State Key Laboratory of Chemical Oncogenomics, School of Chemical Biology and Biotechnology, Peking University Shenzhen Graduate School, Shenzhen, China; 11 Present Address: Shenzhen Bay Laboratory, Shenzhen, China; 12 Present Address: Research Center for Analytical Science and Tianjin Key Laboratory of Biosensing and Molecular Recognition, College of Chemistry, Nankai University, Tianjin, China

**Keywords:** Post-translational modifications, Metabolic pathways

## Abstract

Lysine l-lactylation (K_l-la_) is a novel protein posttranslational modification (PTM) driven by l-lactate. This PTM has three isomers: K_l-la_, *N*-ε-(carboxyethyl)-lysine (K_ce_) and d-lactyl-lysine (K_d-la_), which are often confused in the context of the Warburg effect and nuclear presence. Here we introduce two methods to differentiate these isomers: a chemical derivatization and high-performance liquid chromatography analysis for efficient separation, and isomer-specific antibodies for high-selectivity identification. We demonstrated that K_l-la_ is the primary lactylation isomer on histones and dynamically regulated by glycolysis, not K_d-la_ or K_ce_, which are observed when the glyoxalase system was incomplete. The study also reveals that lactyl-coenzyme A, a precursor in l-lactylation, correlates positively with K_l__-la_ levels. This work not only provides a methodology for distinguishing other PTM isomers, but also highlights K_l-la_ as the primary responder to glycolysis and the Warburg effect.

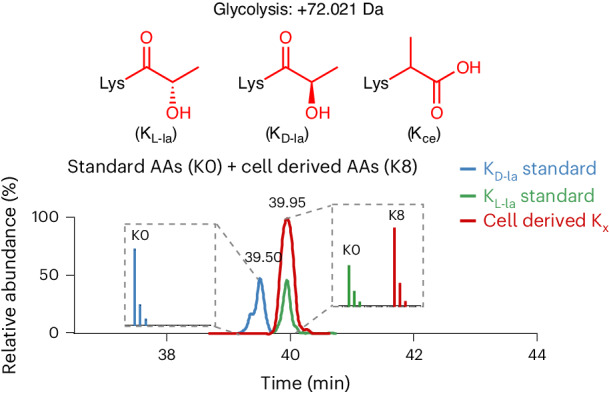

## Abstract (web summary)

Using a combination of antibody- and LC–MS/MS-based methods, Zhang et al. reveal lysine l-lactylation as the key lactylation isomer in cellular histones, responding dynamically to glycolysis and positively correlating with lactyl-CoA levels, providing insights into the Warburg effect.

## Main

Histones undergo a variety of posttranslational modifications (PTMs), which can have substantial effects on chromatin activities^1^. Certain histone PTMs are regulated by cellular metabolites^2–4^, which can either act as cofactors (or precursors) for PTM-catalyzing enzymes^5^ or can directly mediate PTMs that are not added enzymatically^6,7^. For example, acetylation and methylation on lysine are driven by acetyl-coenzyme A (CoA) and *S*-adenosylmethionine, respectively^8,9^, while NAD^+^ and α-ketoglutarate are cofactors for enzymes that remove these marks^8^. These metabolite-sensitive histone PTMs exemplify the interconnection of metabolism and epigenetic regulation^3,9,10^.

We recently discovered a histone PTM called lysine lactylation (originally Kla, but hereafter K_l-la_) that is induced by glycolysis-derived l-lactate^11^. We suggested that lactyl-CoA is the high-energy intermediate connecting l-lactate and K_l-la_ formation. Our research revealed that the acetyltransferase p300 can use chemically synthesized lactyl-CoA as a cofactor for enzymatic histone K_l-la_ formation^11^, while HDAC 1–3 (refs. ^12,13^) as well as SIRT2 (ref. ^14^) can catalyze the lysine delactylation. Although the enzymatic source of lactyl-CoA remains unknown, Varner et al. quantified lactyl-CoA in mammalian cell and tissue samples^15^. Nevertheless, it remains unknown whether other high-energy metabolites contribute to histone K_l-la_ in vivo.

**Fig. 1:**
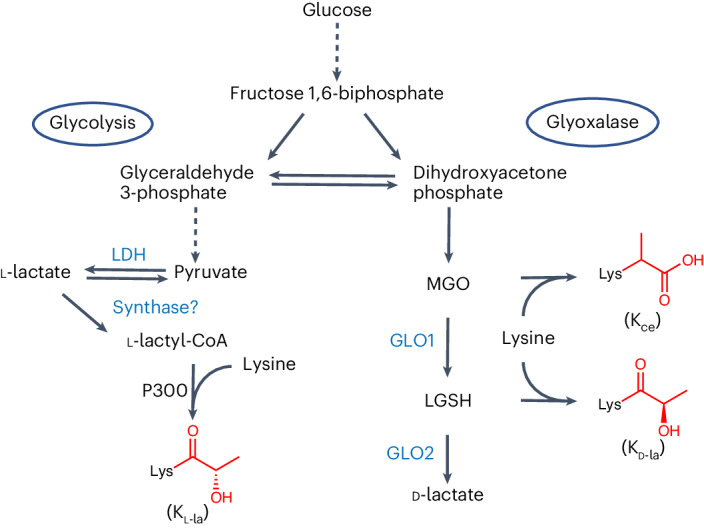
K_l-la_, K_d-la_ and K_ce_ are structural isomers that are associated with glycolysis. A schematic overview delineating the metabolic relationship between glycolysis and the structural isomers K_l-la_, K_d-la_ and K_ce_. It includes the chemical structures of these isomers, emphasizing their distinct molecular configurations and how they are produced through the glycolytic pathway.

After our report on histone K_l-la_, its stereoisomeric modification lysine d-lactylation (originally called lactoylation, but hereafter K_d-la_) was subsequently discovered. K_d-la_ is formed by an uncatalyzed reaction between proteins and *S*-d-lactoylglutathione (LGSH), which is produced through the glyoxalase pathway^16,17^ (Fig. 1). The glyoxalase pathway involves two enzymes: glyoxalase 1 (GLO1) and glyoxalase 2 (GLO2). GLO1 conjugates methylglyoxal (MGO), a byproduct of glycolysis, to glutathione to form LGSH, which is then hydrolyzed by GLO2 to produce d-lactate and regenerate cellular glutathione^18^. MGO is highly reactive (its physiological concentration is about 0.1–2 µM)^19^ and is known to react with a variety of protein residues, including cysteine, arginine and lysine^18^. One of the MGO adducts, *N*-ε-(carboxyethyl)-lysine (K_ce_ hereafter, shown in Fig. 1), has been reported on histones, but at much lower levels than MGO-derived modifications on arginine residues^20^. Due to their identical molecular weights and structural similarity, K_l-la_, K_d-la_ and K_ce_ cannot currently be differentiated by high-performance liquid chromatography with mass spectrometry (HPLC–MS) analysis. Therefore, Brookes and Muller groups raised concerns regarding the identity of Kla modification and presence of lactyl-CoA. They also suggest the direct chemical reaction between lysine’s amine group and MGO and LGSH in cells^21,22^. In addition, it remains unclear whether K_d-la_ and K_ce_ can respond to dynamic changes of cellular glycolysis in a similar fashion as K_l-la_.

To address these fundamental questions, we undertook a thorough investigation to distinguish between the three modifications. Here we report our development and application of multiple orthogonal techniques, including analytical, chemical biological and immunological methods, to differentiate K_l-la_, K_d-la_ and K_ce_. Our results provide compelling evidence for the distinct detection of these three modifications. Our findings reveal that only K_l-la_ is responsive to glycolysis in wild-type cells, corroborating our previous report^11^.

## Results

### PTM-specific antibodies differentiate K_l-la_, K_d-la_ and K_ce_

**Fig. 2:**
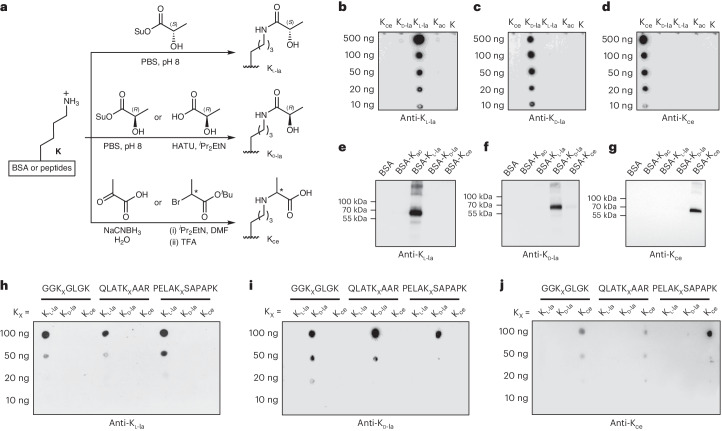
PTM-specific antibodies can distinguish K_l-la_, K_d-la_ and K_ce_. **a**, A schematic diagram illustrating the process of generating PTM-specific compounds used for immunoblotting assays, with detailed procedures available in Methods and Supplementary Information. **b**–**j**, Immunoblots titrating the antibodies’ specificities against various PTM-specific antigens, including synthetic peptide libraries (K_l-la_ (**b**), K_d-la_ (**c**), K_ce_ (**d**)), BSA derivatives (K_l-la_ (**e**), K_d-la_ (**f**), K_ce_ (**g**)) and synthetic sequence-specific histone peptides with the indicated modifications (K_l-la_ (**h**), K_d-la_ (**i**), K_ce_ (**j**)). These blots represent consistent results from at least three independent repetitions. The peptide libraries are structured with 13 residues in the pattern CXXXXKXXXXX, where ‘X’ represents a mixture of 19 amino acids (excluding cysteine), ‘C’ represents cysteine and the sixth position is a lysine that can be either unmodified or modified with K_l-la_, K_d-la_, K_ce_ or K_ac_ (acetylation).

**Extended Data Fig. 1:**
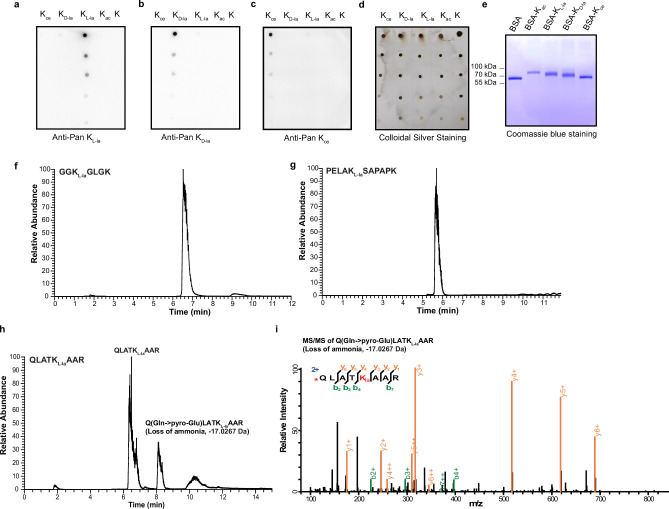
Loading controls and purity assessments for modified BSA and synthesized K_L-la_-containing histone peptides. Related to Fig. 2. **a-d)** Repeated dot blots from Fig. 2b–d, with Colloidal Silver Staining to demonstrate equal loading **(d)**. **e**) SDS-PAGE and Coomassie blue staining showing the purities of unmodified BSA and BSA samples modified with K_L-la_, K_D-la_, K_ce_, or K_ac_. This data represents consistent results from at least three independent repetitions. **f-i**) Chromatography results confirming the purity of synthesized peptides GGK_L-la_GLGK (**f**), PELAK_L-la_SAPAPK (**g**), and QLATK_L-la_AAR (**h**). Notably, in panel **h**, the first peak corresponds to the correctly synthesized peptide QLATK_L-la_AAR, while the second peak suggests ammonia loss from glutamine during peptide storage. **i**) High-resolution MS/MS spectra of the peptide *QLATK_L-la_AAR, showing the fragmentation pattern and confirming the ammonia loss from glutamine as indicated by the second peak in panel **h**. The asterisk preceding ‘Q’ in the peptide sequence denotes this modification.

We first developed immunological reagents that can distinguish K_l-la_, K_d-la_ and K_ce_. We want to point out that the polyclonal pan anti-K_la_ antibodies we generated and used in our original paper^11^ were raised against K_l-la_ instead of K_d-la_ peptides as an antigen. Accordingly, the antibodies recognized K_l-la_ peptides and could detect a dose-dependent increase of K_l-la_ in response to glucose^11^. In this study, we have developed second-generation rabbit monoclonal pan anti-K_l-la_ antibodies, as well as pan anti-K_d-la_ and pan anti-K_ce_ antibodies, for the following experiments. To investigate whether these reagents can distinguish between K_l-la_, K_d-la_ and K_ce_, we performed immunoblotting assays with three sets of PTM-specific antigens (Fig. 2a), including synthetic peptide libraries (Fig. 2b–d and Extended Data Fig. 1a–d), chemically modified bovine serum albumin (BSA) (Fig. 2e–g) and synthetic sequence-specific histone peptides (Fig. 2h–j). The purity and identity of these compounds were confirmed by either SDS–PAGE (Extended Data Fig. 1e) or HPLC with tandem MS (MS/MS) (Extended Data Fig. 1f–i and Supplementary Figs. 1–6). Immunoblotting results showed that pan anti-K_l-la_, pan anti-K_d-la_ and pan anti-K_ce_ antibodies were specific for their corresponding PTMs, with at least 50-fold preference over the other modifications (Fig. 2b–j).

### HPLC separates K_ce_-peptides from K_l-la_ or K_d-la_

**Fig. 3:**
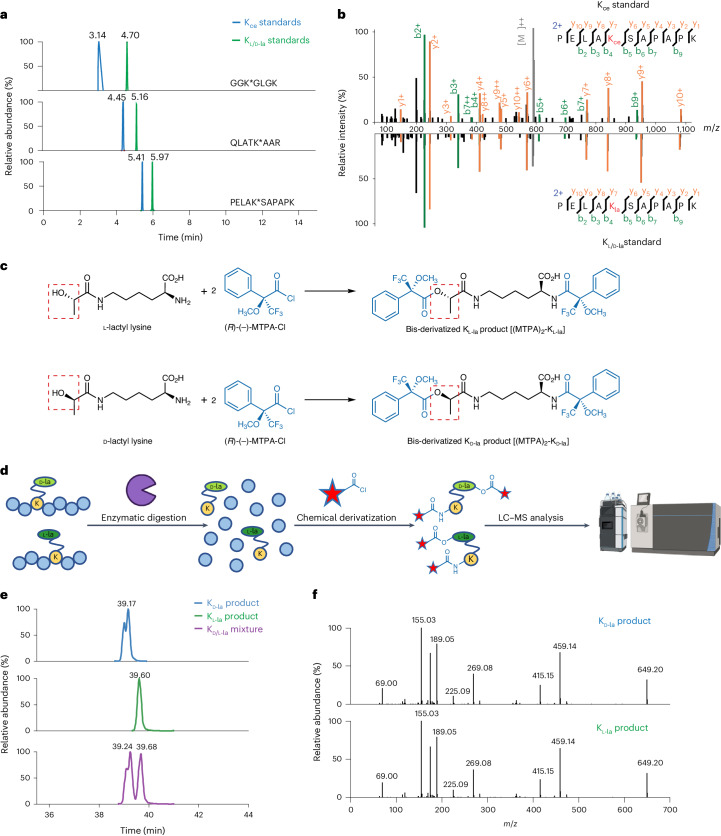
Separating K_l-la_-, K_d-la_- and K_ce_-containing peptides by HPLC–MS/MS. **a**, Extracted ion chromatograms showing the separation of peptides containing K_l-la_, K_d-la_ or K_ce_ modifications. Synthetic peptide standards GGK*GLGK, QLATK*AAR and PELAK*SAPAPK were used, with the asterisk indicating the modified lysine. **b**, High-resolution MS/MS spectra of the PELAK*SAPAPK peptide, modified by either K_ce_ or K_l/d-la_. These spectra reveal that both modifications have the same precursor ion mass and fragmentation patterns. **c**, A schematic diagram illustrating the chemical derivatization reactions between l-lactyl lysine (K_l-la_) or d-lactyl lysine (K_d-la_) and MTPA-Cl, resulting in (MTPA)_2_-K_l-la_ and (MTPA)_2_-K_d-la_ products, respectively. **d**, A workflow diagram showing the process of digesting peptides into individual amino acids, chiral derivatization and subsequent LC–MS/MS analysis for K_l-la_ and K_d-la_. **e**, Extracted ion chromatograms depicting the separation profiles of derivatized K_d-la_, K_l-la_ and their mixtures. **f**, High-resolution MS/MS spectra of derivatization products of K_l-la_ and K_d-la_, showing identical fragmentation patterns.

**Extended Data Fig. 2:**
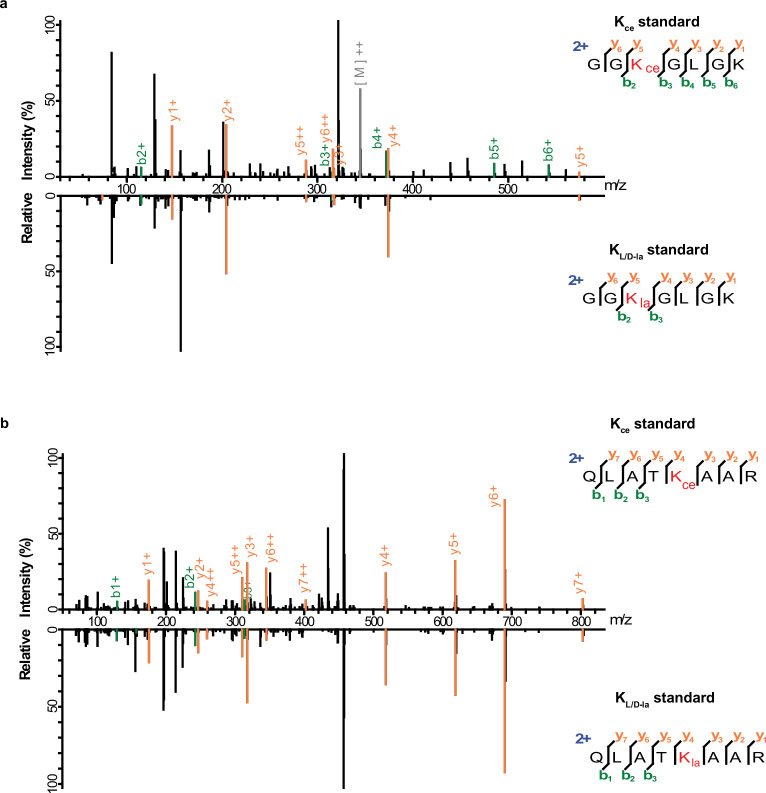
MS/MS spectra of two synthetic K_L/D-la_- and K_ce_- containing histone peptides. Related to Fig. 3. High-resolution MS/MS spectra of the synthetic peptide standards GGK*GLGK (**a**) and QLATK*AAR (**b**), each modified by K_ce_ (**top**) and K_L/D-la_ (**bottom**). These spectra confirm that peptides with these modifications have the same precursor ion mass and display identical MS/MS fragmentation patterns.

To distinguish K_l-la_, K_d-la_ and K_ce_ by orthogonal methods, we performed HPLC experiments. Stereoisomers with different configurations at just one stereocenter, such as K_l-la_ and K_d-la_, are difficult to separate in a typical reversed-phase HPLC column (for example, a C18 column). However, constitutional isomers such as K_l/d-la_ versus K_ce_ can possibly be separated by HPLC, as demonstrated in our previous study on 2-hydroxyisobutyryl lysine isomers^23^. Using three groups of synthetic histone peptides containing one of the three isomers, we demonstrated that although the peptide pairs bearing either K_l-la_ or K_d-la_ could not be separated, they can be easily distinguished from their counterpart bearing K_ce_ by reverse-phase HPLC (Fig. 3a,b and Extended Data Fig. 2a,b).

### Chiral reaction and HPLC for K_l-la_/K_d-la_ separation

Reverse-phase HPLC usually cannot resolve a peptide containing K_l-la_ from its counterpart bearing K_d-la_. We sought to resolve K_l-la_ from K_d-la_ at the single amino acid level. This approach offers an advantage as it enables the comprehensive assessment of all peptides present within the cell without the need to synthesize numerous peptide standards. We hypothesized that it might be possible to separate PTM-containing amino acid pair, l-lactyl lysine and d-lactyl lysine, by HPLC if their difference in stereochemistry could be accentuated by introducing additional chiral chemical group. To test this hypothesis, we took advantage of the classic chiral derivatization reaction using Mosher’s acid chloride (MTPA-Cl), which has been widely used to differentiate chiral molecules and determine their stereo-configurations^24^. As MTPA-Cl can react with both amine and hydroxyl groups, the reaction between MTPA-Cl and the two modified amino acids generates bis-derivatized products, (MTPA)_2_-K_l-la_ and (MTPA)_2_-K_d-la_, respectively. The chiral difference is thus magnified, increasing the likelihood of chromatographic separation (Fig. 3c). To investigate this possibility, we first digested two synthetic peptides containing K_l-la_ or K_d-la_ into individual amino acids with leucine aminopeptidase. The resulting amino acids were derivatized by MTPA-Cl and then analyzed by HPLC–MS/MS (Fig. 3d). We found that (MTPA)_2_-K_l-la_ and (MTPA)_2_-K_d-la_ were well separated (Fig. 3e) and have the same fragmentation pattern in MS/MS (Fig. 3f). The above data demonstrate a proof of concept for distinguishing K_l-la_, K_d-la_ and K_ce_ by HPLC–MS.

### K_l-la_ is predominant on histones, not K_d-la_ or K_ce_

**Fig. 4:**
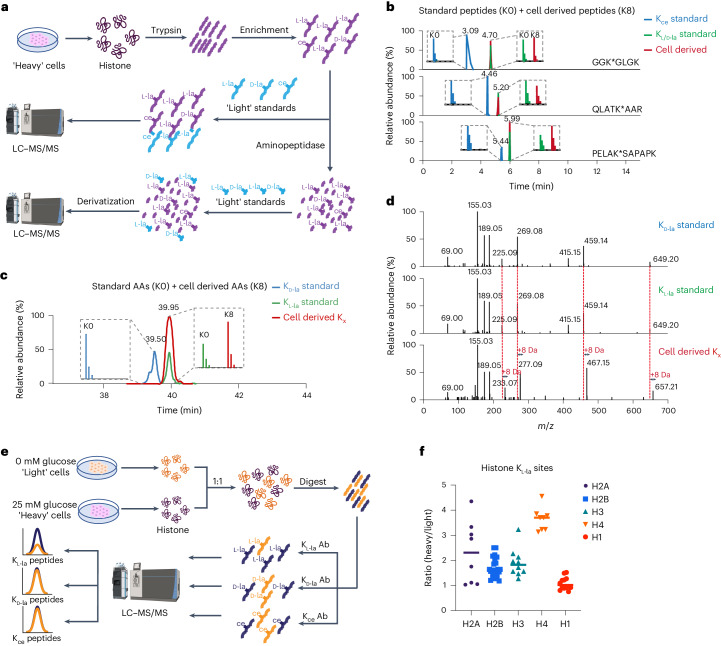
K_l-la_ is the prevalent PTM on cellular histones. **a**, A workflow for analyzing K_l-la_, K_d-la_ and K_ce_ on cellular histones. Histones were extracted from cultured human MCF-7 cells, which were labeled with heavy isotopes (K8 or l-Lys-^13^C_6_, ^15^N_2_). These histones were digested with trypsin, and the resulting peptides containing the target PTMs were enriched using a mixture of pan anti-K_l-la_, pan anti-K_d-la_ and pan anti-K_ce_ antibodies to enhance detection sensitivity. **b**, The enriched peptides were spiked with light standard peptides (K0 or l-Lys-^12^C_6_,^14^N_2_) and analyzed by LC–MS/MS. The standard peptide sequences are GGK*GLGK, QLATK*AAR and PELAK*SAPAPK, with the asterisk marking the modified lysine residue. The extracted ion chromatograms indicate the presence of K_l/d-la_ modifications but not K_ce_ in the cell-derived histone peptides. The coelution of cell-derived peptides with the modified standards confirms the presence of K_l/d-la_. **c**, The enriched peptides were further digested into individual amino acids (cell-derived AAs, K8), spiked with light K_l-la_ and K_d-la_ standards (standard AAs, K0), and derivatized with MTPA-Cl before undergoing LC–MS/MS analysis. The extracted ion chromatograms reveal K_l-la_ is present in the cell-derived histone peptides, while K_d-la_ is absent. **d**, High-resolution MS/MS spectra verifying identical fragmentation patterns between cell-derived K_L-la_ and synthetic light standards, with an 8 Da mass shift due to the heavy isotopic lysine backbone. **e**, A workflow for analyzing histone K_l-la_, K_d-la_ and K_ce_ in response to glycolysis. MCF-7 cells were cultured under different glucose conditions and labeled with isotopes. Histones were extracted and digested and the peptides containing the PTMs were enriched separately and quantified using LC–MS/MS. **f**, LC–MS/MS quantification results indicate that a high glucose concentration induced K_l-la_ on most core histones sites (H2A, H2B, H3 and H4), but not on linker histone H1. The heavy to light ratio was normalized to protein abundance.

**Extended Data Fig. 3:**
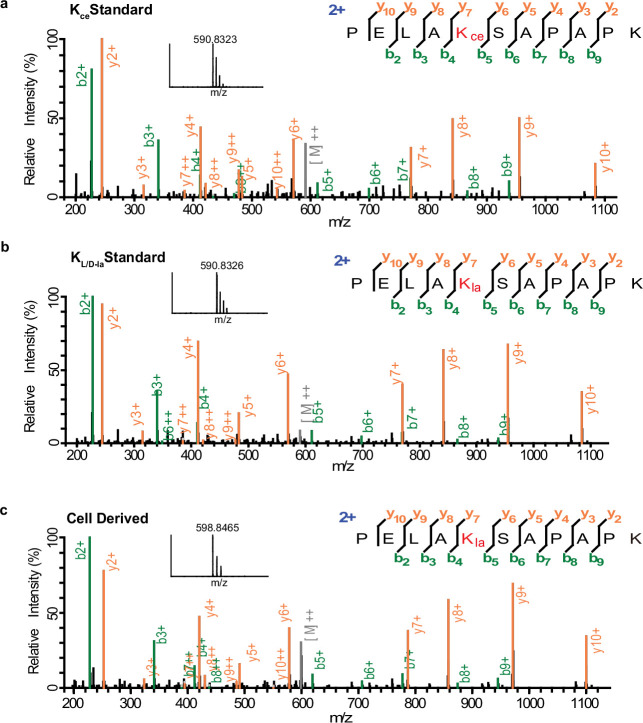
Identification and verification of a K_L/D-la_-containing histone H2B peptide. Related to Fig. 4. The high-resolution MS/MS spectrum in panel **a** presents a cell-derived histone peptide, PELAK_la_SAPAPK, labeled with heavy isotopes (L-Lys-^13^C_6_, ^15^N_2_). This cell-derived peptide is compared against synthetic standard peptides containing K_ce_ or K_L/D-la_, labeled with light isotopes (L-Lys-^12^C_6_, ^14^N_2_), as shown in panels **b** and **c**, respectively. The comparison reveals that both the cell-derived peptide and the synthetic peptides with the modifications of interest exhibit similar MS/MS fragmentation patterns and precursor ion masses. The only difference observed is the mass shift attributable to the isotope labeling. The specific m/z values of the precursor ions are displayed in the insets of the spectra.

**Extended Data Fig. 4:**
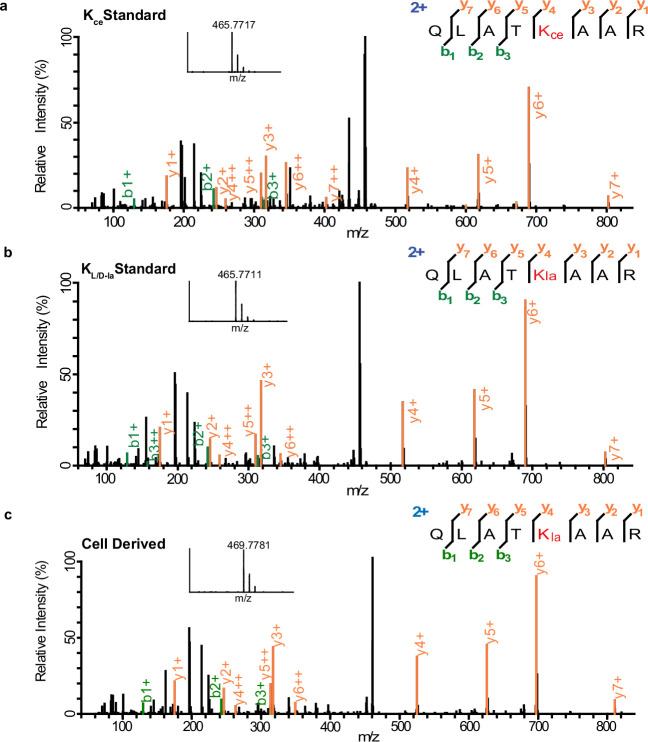
Identification and verification of a K_L/D-la_-containing histone H3 peptide. Related to Fig. 4. The high-resolution MS/MS spectrum in panel **a** presents a cell-derived histone peptide, QLATK_la_AAR, labeled with heavy isotopes (L-Lys-^13^C_6_, ^15^N_2_). This cell-derived peptide is compared against synthetic standard peptides containing K_ce_ or K_L/D-la_, labeled with light isotopes (L-Lys-^12^C_6_, ^14^N_2_), as shown in panels **b** and **c**, respectively. The comparison reveals that both the cell-derived peptide and the synthetic peptides with the modifications of interest exhibit similar MS/MS fragmentation patterns and precursor ion masses. The only difference observed is the mass shift attributable to the isotope labeling. The specific m/z values of the precursor ions are displayed in the insets of the spectra.

**Extended Data Fig. 5:**
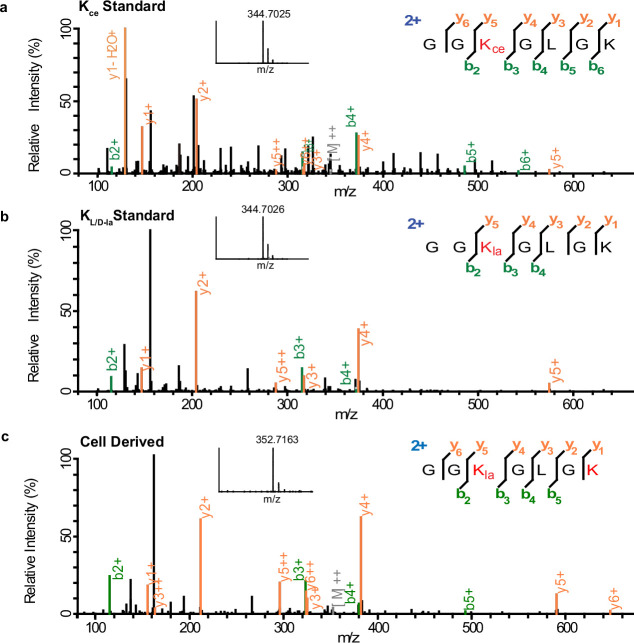
Identification and verification of a K_L/D-la_-containing histone H4 peptide. Related to Fig. 4. The high-resolution MS/MS spectrum in panel **a** presents a cell-derived histone peptide, GGK_la_GLGK, labeled with heavy isotopes (L-Lys-^13^C_6_, ^15^N_2_). This cell-derived peptide is compared against synthetic standard peptides containing K_ce_ or K_L/D-la_, labeled with light isotopes (L-Lys-^12^C_6_, ^14^N_2_), as shown in panels **b** and **c**, respectively. The comparison reveals that both the cell-derived peptide and the synthetic peptides with the modifications of interest exhibit similar MS/MS fragmentation patterns and precursor ion masses. The only difference observed is the mass shift attributable to the isotope labeling. The specific m/z values of the precursor ions are displayed in the insets of the spectra.

Next, we investigated which out of K_L-la_, K_d-la_ and K_ce_ is most common as a histone mark in cells. To improve the sensitivity and accuracy of the assay, we first cultured human MCF-7 cells in ‘heavy’ media (containing K8 [^13^C_6_, ^15^N_2_] heavy isotope-labeled l-lysine) and enriched cellular histone peptides bearing the PTMs of interest using pan anti-K_l-la_, pan anti-K_d-la_ and pan anti-K_ce_ antibodies. These enriched ‘heavy’ PTM-containing peptides were then mixed with synthetic ‘light’ (K0 [^12^C_6_, ^14^N_2_] light isotope-labeled l-lysine) K_l-la_-, K_d-la_- and K_ce_-containing peptide standards for subsequent HPLC–MS/MS analysis (Fig. 4a). The synthetic peptide standards consist of three sets of modified histone peptides with sequences of interest: GGK*GLGK (histone H4), QLATK*AAR (histone H3) and PELAK*SAPAPK (histone H2B). In these sequences, K* represents one of the following modifications: K_l-la_, K_d-la_ or K_ce_. Our HPLC–MS/MS analysis showed that the cell-derived peptides exhibited the same HPLC retention time and MS/MS fragmentation patterns as the K_l/d-la_ standard peptides (Fig. 4b and Extended Data Figs. 3–5). However, their retention time did not align with the K_ce_ standard peptides (Fig. 4b). These findings strongly indicate that K_l/d-la_ but not K_ce_ is the predominant modification form on histones.

To investigate whether lactylation was present in the K_l-la_ or K_d-la_ form, we conducted a series of experiments. First, we digested the affinity-enriched ‘heavy’ peptides into single amino acid residues with leucine aminopeptidase. We then spiked in with ‘light’ K_l-la_ and K_d-la_ amino acid standards and performed chiral derivatization with MTPA-Cl (Fig. 4a). The chiral derivatization reaction introduced an additional chiral functional group that increases the stereochemical difference between K_l-la_ and K_d-la_. The resulting reaction products were analyzed by HPLC–MS/MS. Our results showed that the reaction products derived from the cell samples coeluted with the K_l-la_ reaction products but not with the K_d-la_ reaction products (Fig. 4c,d). These results provide clear evidence that the predominant form of histone lactylation is the l form rather than the d form.

To corroborate these results, we carried out SILAC (stable isotope labeling by amino acids in cell culture)-based quantitative MS analysis on histone K_l-la_, K_d-la_ and K_ce_. In this experiment, human MCF-7 cells were cultured in ‘light’ and ‘heavy’ media for six cell doublings. The ‘light’ cells were treated with no glucose, while the ‘heavy’ cells were treated with high glucose for 24 hours. Following the treatment, equal numbers of the cells from both conditions were combined and subjected to histone extraction. The resulting histone samples were then digested into peptides using trypsin and subsequently enriched for each type of PTM-containing peptides using their corresponding PTM-specific antibodies (Fig. 4e). These experiments identified 62 K_l-la_ modified histone peptides that were induced by high glucose (Fig. 4f). We noted an intriguing observation that K_l-la_ on core histones, rather than linker histones, displayed a markedly more pronounced response to high glucose conditions. However, further studies are required to fully understand the underlying reasons for this observed difference. In contrast, under our experimental conditions, we did not detect any peptides bearing K_d-la_ or anti-K_ce_, when using anti-K_d-la_ or anti-K_ce_ antibody for enriching their corresponding peptides. Our results indicate that K_d-la_ and K_ce_ modifications on histones were not detectable from cells cultured in either low or high glucose. This result is consistent with previous studies showing that K_d-la_ or K_ce_ are not present on histones in wild-type cells^16,20^.

**Extended Data Fig. 6:**
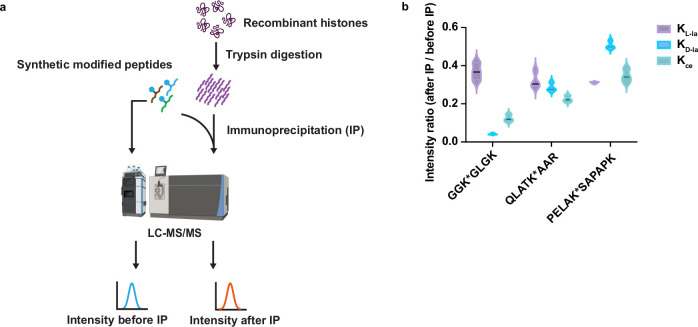
Assessment of the enrichment capabilities of the three antibodies. Related to Fig. 4. **a)** An illustration depicting the experimental setup for assessing antibody enrichment. In each immunoprecipitation (IP) experiment, a trace amount (0.3 ng) of three synthetic peptides (GGK*GLGK, QLATK*AAR, and PELAK*SAPAPK) were mixed with a larger quantity of recombinant histone peptides (100 µg). The standard IP-LC-MS/MS procedure was followed, and the peptide intensities were measured to calculate the ratio of intensities ‘after IP’ compared to ‘before IP’. Panel **b)** displays the resulting intensity ratios for the three modified peptides, which indicate the relative enrichment performance of the antibodies.

In the above assays, we used antibody-based enrichment to increase the sensitivity for PTM detection. To further increase the rigor of these assays, we assessed the enrichment capability of the three antibodies by semiquantifying the intensities of three spiked-in modified peptides both before and after enrichment by each antibody (Extended Data Fig. 6a). Our data demonstrated that all three antibodies can efficiently enrich a small number of modified peptides among a large number of unmodified peptides, as evidenced by the spiking in of three sets of modified histone peptides (from H3, H4 and H2B, respectively) (Extended Data Fig. 6b). It is noteworthy that, unlike K_l-la_, we were unable to detect any of these three peptides or any other modification sites on cellular histones bearing either K_d-la_ or K_ce_. Therefore, we propose that K_l-la_, but not K_d-la_ or K_ce_, represents the main isomer of the three PTMs on cellular histones. However, we cannot exclude the possibility that histone K_d-la_ and K_ce_ are present in cells, but their abundance was too low to be detected in our assays. Histone marks with low stoichiometries could potentially play significant roles in locus-specific regulation, such as H3K4 trimethylation, while it is less plausible for low-level nonnuclear PTMs to have a significant impact, especially in context of inhibiting an enzyme’s function.

In summary, our data showed that K_l-la_ is the dominant isomer among the three structural isomers of lysine lactylation on cellular histones.

### Glycolysis differentially regulates K_l-la_, K_d-la_ and K_ce_

**Fig. 5:**
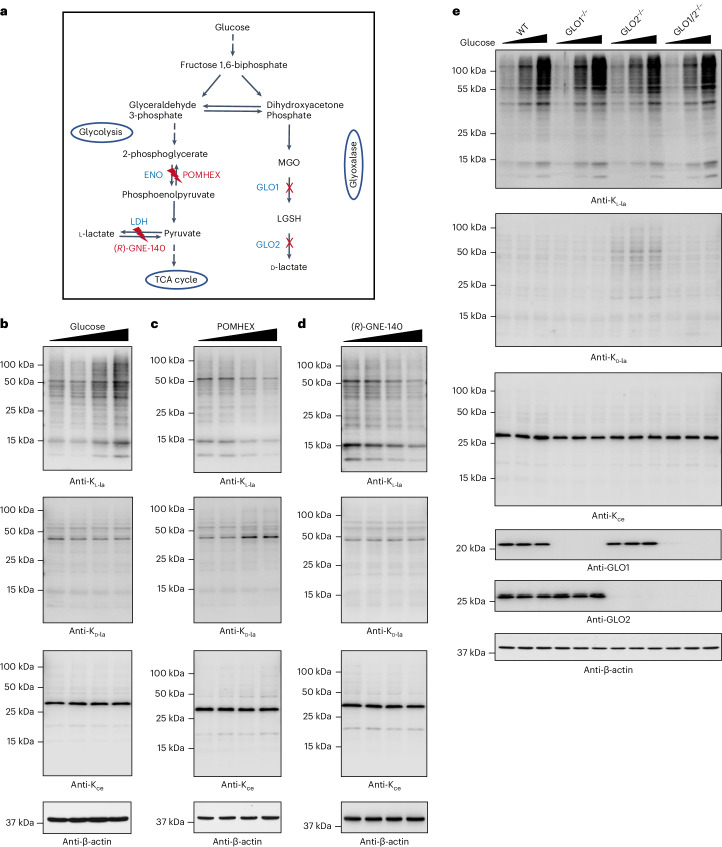
Differential regulation of global K_l-la_, K_d-la_ and K_ce_ by glycolysis. **a**, A schematic of enzymes and inhibitors that influence metabolite production related to these modifications, including ENO, LDH, GLO1 (glyoxalase 1), GLO2 (glyoxalase 2), POMHEX (ENO inhibitor) and (*R*)-GNE-140 (LDH inhibitor). **b**–**e**, Western blots showing the effects of varying glucose concentrations (**b**), POMHEX (**c**), (*R*)-GNE-140 (**d**) and glyoxalase deletion (**e**) on these modifications in MCF-7 cells and HEK293T cells. The following treatments were used: glucose 0.1, 1, 5 and 25 mM (**b**); POMHEX 0, 0.1, 0.5 and 2.5 µM (**c**); (*R*)-GNE-140 0, 0.5, 2.5 and 10 µM (**d**) and glucose 1, 5 and 25 mM (**e**). β-Actin served as a control across all blots. Results are consistent across three independent experiments. Source data

**Extended Data Fig. 7:**
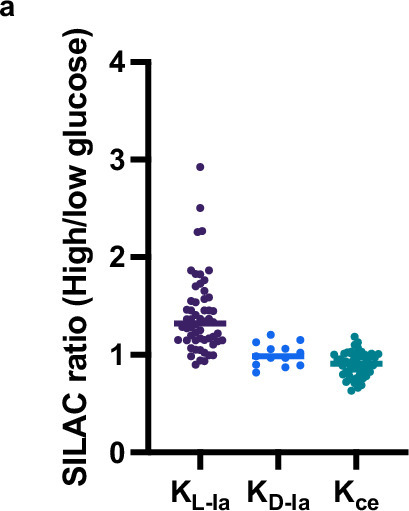
Global alterations in K_L-la_, K_D-la_, and K_ce_ in response to glycolysis. Related to Fig. 5. **a)** Cell cultures exposed to low glucose levels (0.1 mM glucose) and high glucose levels (25 mM glucose) were combined. The resulting whole protein lysates were extracted, digested, and specific peptides modified with K_L-la_, K_D-la_, and K_ce_ were selectively enriched using targeted antibodies. The enriched peptides were quantified through SILAC (Stable Isotope Labeling with Amino acids in Cell culture)-assisted LC-MS/MS analyses.

K_l-la_, K_d-la_ and K_ce_ are all formed from precursors that are intermediates in glycolysis (Fig. 5a). Nevertheless, how the three PTMs are dynamically regulated in response to changes in glycolysis has not been systematically studied. To this end, we first cultured human MCF-7 cells in media containing different glucose concentrations and examined the cellular levels of these PTMs by western blot. We found that global K_l-la_ levels, on both histones and nonhistone proteins, were induced by glucose in a dose-dependent manner (Fig. 5b). In contrast, global K_d-la_ and K_ce_ levels were largely unresponsive to extracellular glucose concentrations (Fig. 5b). To further validate this western blot result, we quantify the global levels of these three isomeric modifications in whole-cell protein lysates using LC–MS/MS after exhaustive removal of small molecules. For this purpose, we cultured MCF-7 cells in both ‘light’ and ‘heavy’ Dulbecco’s modified Eagle’s medium (DMEM) media, for low glucose conditions (0.1 mM) and high glucose conditions (25 mM), respectively. The whole-cell protein lysates were generated from mixed cells (with equal numbers of ‘light’ and ‘heavy’ cells) and digested with trypsin. The resulting peptides were subjected to affinity enrichment to isolate the peptides bearing one of the three PTMs of interest using its corresponding antibody. The results from SILAC-based proteomic quantification revealed that under high glucose conditions, most K_l-la_ modified peptides exhibited an increase, while most K_d-la_ and K_ce_ modified peptides showed no apparent alteration compared to low glucose conditions (Extended Data Fig. 7a). These findings were consistent with our western blot data.

Furthermore, we perturbed glycolysis by inhibiting enolase (ENO) or lactate dehydrogenase (LDH) (Fig. 5a). We expected that inhibition of ENO activity by POMHEX^25^ (which was also suggested in Muller’s comments^22^) would result in the entry of glucose-derived carbon into the glyoxalase pathway and simultaneously block lactate production. In contrast, inhibition of LDH activity may only abolish l-lactate production but not affect the glyoxalase pathway (Fig. 5a). We found that global K_l-la_ levels were decreased by POMHEX in a dose-dependent manner, while K_d__-la_ levels were increased and K_ce_ levels were marginally affected (Fig. 5c). In contrast, (*R*)-GNE-140 (ref. ^26^), an inhibitor of LDH, reduced global K_l-la_ levels without affecting K_d-la_ or K_ce_ levels (Fig. 5d).

**Fig. 6:**
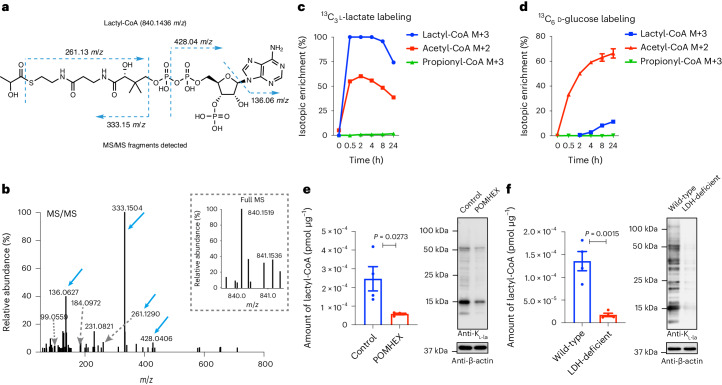
Lactyl-CoA is positively correlated with K_l-la_ in response to glycolysis. **a**, The chemical structure of lactyl-CoA with four MS2 fragments identified through high-resolution nano-HPLC–MS/MS analysis. **b**, The tandem mass spectrum of lactyl-CoA from HepG2 cells is presented, showing characteristic fragmentation ions and the precursor ion with a molecular weight of 840.1519 Da. **c**,**d**, Incorporation of ^13^C_3_ l-lactate (**c**) and ^13^C_6_ d-glucose (**d**) into lactyl-CoA and other metabolites in HepG2 cells. **e**,**f**, Lactyl-CoA concentrations were measured using LC–MS/MS, and global K_l-la_ levels were determined via western blots in wild-type HepG2 cells with or without 2.5 µM POMHEX treatment (**e**), as well as in HepG2 cells with a deletion of both LDHA and LDHB (**f**). *n* = 4 biological replicates. The data are presented as mean values ± s.e.m. Statistical significance was determined using a two-tailed Student’s *t*-test. Source data

**Extended Data Fig. 8:**
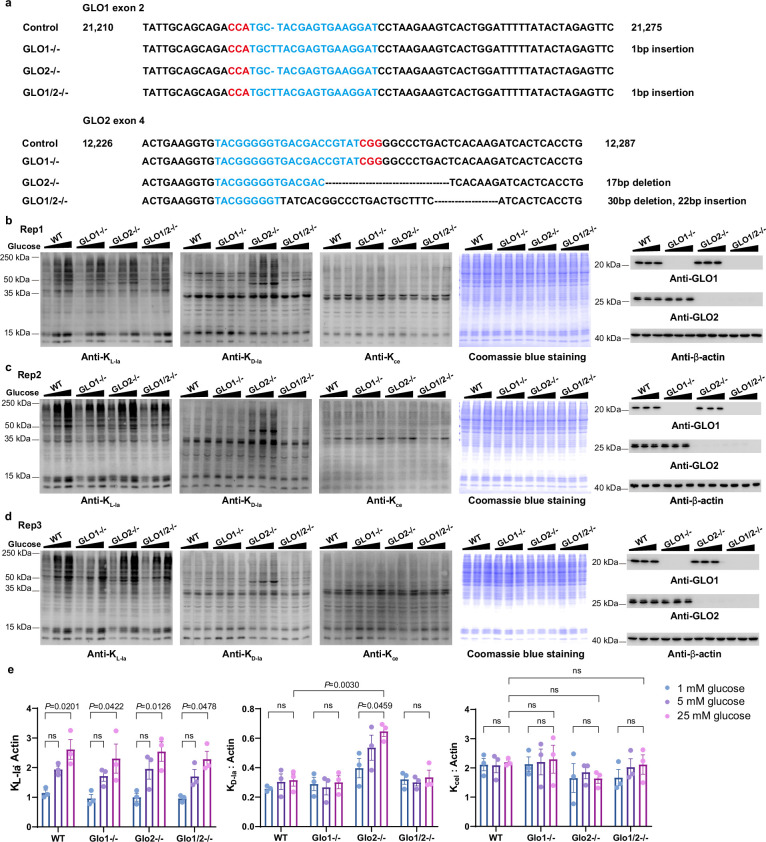
Global changes in K_L-la_, K_D-la_, and K_ce_ in cells deficient in glyoxalase enzymes. Related to Fig. 5. **a)** GLO1−/− cells are produced by a one-base insertion in GLO1’s exon 2, while GLO2−/− cells result from a 17-base deletion in GLO2’s exon 4. GLO1/2 double-deficient cells (GLO1/2−/−) are created by combing mutations including a one-base insertion in GLO1’s exon 2 and both a 30-base deletion and a 22-base insertion in GLO2’s exon 4. The guide RNA (gRNA) target sites are highlighted in blue with PAM (protospacer adjacent motif) sites in red. **b-d)** Triplicates of the Western blot experiments corresponding to Fig. 5e, using β-Actin as a loading control. **e)** The statistical analysis derived from densitometry measurements of the blots shown in panels **b-d**. Data are presented as mean values +/− SEM. Statistical significance was determined using two-way analysis of variance (ANOVA) followed by Tukey’s test for multiple comparisons. Source data

The glyoxalase pathway is one of the most efficient systems for removing chemically reactive by-products such as MGO and LGSH from cellular metabolism^18^. We next investigated whether K_l-la_, K_d-la_ and K_ce_ are regulated by the glyoxalase pathway. We generated GLO1-, GLO2- and GLO1/2-deficient human embryonic kidney (HEK) 293T cells using the CRISPR–Cas9 technology (Extended Data Fig. 8a). After culturing these cells in media containing different glucose concentrations, we found that global K_l-la_ was responsive to glucose concentration, but not regulated by glyoxalases (Fig. 5e and Extended Data Fig. 8b–e). In contrast, global K_d-la_ was induced in the absence of GLO2, but it exhibited much lower sensitivity to changes in glucose concentration (Fig. 6e and Extended Data Fig. 8b–e). We did not observe an increase in global K_ce_ levels in the absence of GLO1 (Fig. 6e and Extended Data Fig. 8b–e). It is possible that alternative pathways, such as aldose reductase, contribute to the removal of MGO in the absence of GLO1 (ref. ^27^). It has been observed that cells deficient in GLO1 and cultured in high glucose media did not accumulate K_ce_ or arginine adduct modifications due to compensatory mechanisms^28^.

Together, these data suggest that K_l-la_, K_d-la_ and K_ce_ are differently regulated during glycolysis, and that the glyoxalase pathway prevents the formation of K_d-la_ and K_ce_ in cells without affecting glycolysis-sensitive K_l-la_.

### Lactyl-CoA correlates with K_l-la_ in response to glycolysis

We previously proposed the existence of lactyl-CoA as a cofactor for cellular K_l-la_ formation^11^. Some short-chain acyl-CoA synthetases are known to produce not only acetyl-CoA from acetate, but also propionyl-CoA from propionate^29^ and crotonyl-CoA from crotonate^30^. Intracellular l-lactate is very abundant, and therefore it is possible that lactyl-CoA is formed by a similar mechanism. Supporting this hypothesis, Varner et al. recently showed the existence of lactyl-CoA in mammalian cells and tissues^15^. Further, we demonstrated that acyltransferase p300 can use chemically synthesized lactyl-CoA as a cofactor for the in vitro enzymatic histone K_l-la_ reaction^11^.

To test our hypothesis, we first confirmed the existence of lactyl-CoA in cultured cells (Fig. 6a,b). To examine the metabolic turnover of lactyl-CoA, we carried out isotope tracing experiments using ^13^C_3_-l-lactate and U-^13^C_6_-d-glucose, respectively. We found that both lactyl-CoA and acetyl-CoA were labeled very quickly by exogenous ^13^C_3_-l-lactate, with decreases in labeling after 4 hours (Fig. 6c). The decrease in isotope enrichment was probably due to dilution by ^12^C_3_ lactyl-CoA and ^12^C_2_ acetyl-CoA generated endogenously. We also noticed that maximal labeling of lactyl-CoA (near 100%) was much higher than that of acetyl-CoA (50–60%) (Fig. 6c). This may have occurred because l-lactate contributes differently to formation of lactyl-CoA and acetyl-CoA, or because the two acyl-CoAs have different half-lives in cells. Propionyl-CoA, which is not derived directly from l-lactate metabolism, was not isotopically labeled by ^13^C_3_-l-lactate under the same conditions (Fig. 6c).

Unlike ^13^C_3_-l-lactate, treating cells with U-^13^C_6_-d-glucose resulted in labeling of acetyl-CoA much faster than lactyl-CoA (Fig. 6d). This difference correlates well with our previous experiment in which histone K_l-la_ was labeled far more slowly than histone lysine acetylation (K_ac_) by U-^13^C_6_-d-glucose^11^. Under the same conditions, we did not observe any labeling of propionyl-CoA by U-^13^C_6_-d-glucose (Fig. 6d). Together, these data demonstrate that lactyl-CoA can be metabolically derived from both l-lactate and glucose.

**Extended Data Fig. 9:**
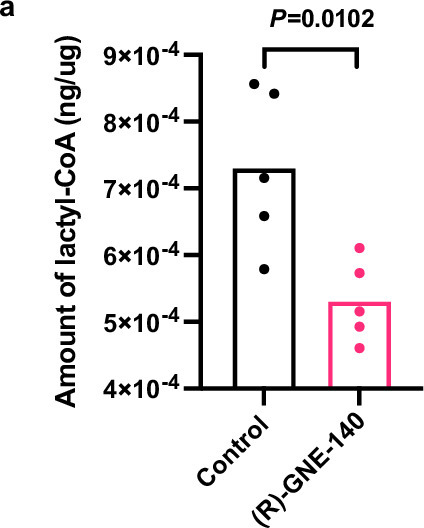
Quantitative analysis of lactyl-CoA in cells treated with (R)-GNE-140. Related to Fig. 6. **a)** Lactyl-CoA concentrations were quantified in wild-type HepG2 cells using LC-MS/MS, with or without treatment by 10 µM (R)-GNE-140. The experiment was conducted with n = 5 biological replicates, and the data were normalized to protein content. The Data are presented as mean values +/− SEM. Statistical significance was determined using a two-tailed Student’s *t*-test.

To examine whether lactyl-CoA is regulated by glycolysis and correlates with K_l-la_ levels, we measured lactyl-CoA in cells where ENO or LDH was blocked. We observed that lactyl-CoA levels were much lower on POMHEX treatment (Fig. 6e). Similarly, in LDH-deficient cells or cells treated with (*R*)-GNE-140, lactyl-CoA levels were much lower than those in control cells (Fig. 6f and Extended Data Fig. 9a). These data demonstrated that lactyl-CoA is dynamically regulated by glycolysis and is positively correlated with K_l-la_ levels (Fig. 6e,f).

In summary, we have successfully distinguished between the isomers K_l-la_, K_d-la_ and K_ce_ using both immunological and analytical chemical methods. Our results clearly demonstrate that, although all three modifications are associated with the glycolytic pathway, K_l-la_ is the dominant modification on cellular histones and the one that responds to changes in glycolysis.

## Discussion

In this study, we used a combination of analytical and immunological methods to distinguish three isomeric protein modifications, K_l-la_, K_d-la_ and K_ce_. Our data suggest that K_l-la_ is the prevalent isomer on cellular histones, and is responsive to glycolysis. In contrast, we could not detect K_d-la_ or K_ce_ on histones by highly sensitive mass spectrometric analysis, even after enrichment. Our results corroborate those of Gaffney et al., who reported that the K_d-la_ modification is enriched on glycolytic enzymes, but not on nuclear proteins^16^. The K_ce_ modification has been reported on histones, but only in GLO1-deficient cells treated with exogenous MGO, but not in wild-type cells^20^. The different distributions of the three PTMs on histones may be explained by their biochemical mechanisms. K_l-la_, and in particular histone K_l-la_, is thought to be primarily a modification catalyzed by acyltransferases (for example, p300) with lactyl-CoA as a cofactor^11^. Like K_ac_, K_l-la_ is enriched on the N-terminal tails of core histones and in gene promoters throughout the genome^11^. In contrast, K_d-la_ and K_ce_ are produced by nonenzymatic reactions as a result of chemically reactive glycolytic by-products escaping from the cellular detoxification system. Since LGSH and MGO, the chemicals responsible for the production of K_d-la_ and K_ce_, respectively, are mainly produced in the cytosol, it makes sense that these two PTMs occur more frequently on cytosolic proteins that are exposed to these active compounds.

Unlike MGO and LGSH, l-lactate is largely a chemically inert molecule. Therefore, we proposed that lactyl-CoA is generated from l-lactate and acts as a high-energy intermediate for K_l-la_ formation^11^, analogous to the relationship between acetyl-CoA and K_ac_. Although we here and others previously^15^ have confirmed the existence of lactyl-CoA in cells, these studies have not addressed the chirality of the detected lactyl-CoA. We argue that lactyl-CoA is in the l form, because it can be labeled by isotopic l-lactate and glucose, and because l-lactate is the major enantiomer in cells (present in millimolar concentration^31^ compared to about 10 µM for d-lactate^32^). However, it is unknown whether d-lactyl-CoA can be formed from d-lactate in lower concentrations. Notably, the Galligan group recently reported that LGSH can undergo conversion to d-lactyl-CoA through a spontaneous S-to-S acyl transfer reactions, contributing to nonenzymatic lactylation (K_d-la_) in GLO2-deficient cells^17^. In addition, several interesting questions about l-lactyl-CoA remain to be investigated, such as how intracellular l-lactyl-CoA is produced and whether l-lactyl-CoA has any other metabolic functions independent of K_l-la_ formation.

The results presented here and in our previous study^11^ clearly demonstrate that the histone lysine lactylation we initially identified is actually K_l-la_. This conclusion is supported by multiple lines of evidence: in wild-type cells, (1) histone K_la_ can be labeled and induced by isotopic l-lactate as we previously reported^11^; (2) histone K_l-la_ is responsive to L-lactate and glucose metabolism^11^; (3) K_D-la_ and K_ce_ cannot be detected on histones through affinity enrichment in combination with high-sensitivity MS; (4) the cellular l-lactate level is much higher than those of MGO, LGSH or d-lactate; (5) lactyl-CoA is well correlated with l-lactate and K_l-la_, and is regulated by glycolysis and (6) histone K_l-la_ is enriched in specific genomic regions rather than randomly distributed on chromatin^11^. The last observation strongly supports that K_l-la_ is caused by an enzyme-mediated process and not uncatalyzed chemical reactions such as the formation of K_d-la_ and K_ce_ from LGSH and MGO, because the chemical reaction could be more random among the genomic regions. Since 2019, several enzymes including P300 (ref. ^11^), CBP^33^, MOF^34^ and YiaC^35^, among others, have been identified to possess lactyltransferase activity. Understanding how these enzymes coordinate to regulate histone K_l-la_ under physiological conditions will be essential for future studies. Together, this study clearly addresses possible confusion in the research community about which of the three structural isomers, K_l-la,_ K_d-la_ or K_ce_, is responsible in glycolysis-derived lactylation.

Differentiation of structural isomers of a PTM is daunting. Such structural isomers are possible not only for K_la_ but also for other modifications. For example, β-hydroxybutyrylation theoretically can be present in two isomers, d-β-hydroxybutyrylation and l-β-hydroxybutyrylation, which could be generated from d-β-hydroxybutyryl-CoA and l-β-hydroxybutyryl-CoA, respectively^36^. Here we report two independent bioanalytical methods for separation of the constitutional and stereochemical isomers of a PTM. We believe that the same approaches can be used to solve other challenges in differentiating and quantifying PTM structural isomers. Given that these chiral modifications are driven by distinct chiral metabolites in cells and they may be regulated by enzymes in different ways, as well as recognized by diverse recognition proteins (or ‘readers’), the ability to differentiate between stereoisomers of PTMs holds significant importance^36^.

## Methods

### Reagents

Pan anti-K_L-la_ (PTM-1401RM, 1:2,000 dilution for western blotting), pan anti-K_D-la_ (ZC288, 1:1,500 dilution for western blotting) and pan anti-K_ce_ (PTM-1701RM, 1:1,000 dilution for western blotting) antibodies were from PTM Biolabs, Inc.; anti-β-actin (no. 4970, 1:6,000 dilution for western blotting) and anti-histone H3 (no. 4499, 1:2,000 dilution for western blotting) were purchased from Cell Signaling Technology, Inc.; anti-GLO1 (A4329, 1:1,000 dilution for western blotting) was purchased from ABclonal; anti-GLO2 (12146-RP02, 1:1,000 dilution for western blotting) was purchased from Sino Biological; d-glucose (G7021), leucine aminopeptidase (L5006) and (*R*)-(−)-MTPA-Cl (65363) were purchased from MilliporeSigma; POMHEX (HY-131904) and (*R*)-GNE-140 (HY-100742A) were purchased from MedChemExpress; [^13^C_6_,^15^N_2_]-vitamin B5 (no. 5065) was purchased from Isosciences; ^13^C_3_-sodium l-lactate (CLM-1579-PK) and U-^13^C_6_-d-glucose (CLM-1396-1) were purchased from Cambridge Isotope Laboratories.

### Chemical synthesis

#### Synthesis of acetyl *N*-hydroxysuccinimide ester

*N*-hydroxysuccinimide (1.15 g, 10 mmol) was added to acetic anhydride (10 ml, 106 mmol), and the reaction was stirred at room temperature for 12 h. The mixture was filtered, the crystalline residue was extracted with hexane and reduced to dryness, and crude product was obtained. The crude product was confirmed by MS and used for chemical acetylation without further purification.

#### Synthesis of l (or d)-lactyl *N*-hydroxysuccinimide ester

l-lactic acid (0.9 g, 10 mmol) and *N*-hydroxysuccinimide (1.38 g, 12 mmol) were dissolved in dichloromethane and stirred at room temperature for 0.5 h. Then, *N*,*N*'-dicyclohexylcarbodiimide (DCC) (2.27 g, 11 mmol) was added and stirring was continued at room temperature overnight. The resulting mixture was filtered, then the organic solution was washed with saturated sodium bicarbonate and brine, and then dried with anhydrous Na_2_SO_4_. The solvent was evaporated and the crude product was obtained. The crude product was confirmed by MS and used for chemical lactylation without further purification.

#### Chemical acetylation, lactylation and *N*-ε-carboxyethylation

The procedures for chemical acetylation, lactylation and *N*-ε-carboxyethylation of BSA are described below. To prepare acetylated BSA, 0.1 mM acetyl *N*-hydroxysuccinimide ester was added to 0.5 mg of BSA (in 500 µl of PBS, pH 8.0). The pH of the solution was adjusted to 8–9 by adding 1 M NaOH. After 1 h, the reaction mixture was centrifuged in a 3 kDa ultrafiltration tube (12,000*g*, 4 min), and washed three times with PBS. Then the acetylated BSA was recovered and dissolved in PBS to a concentration of 2.5 mg ml^−1^. l- and d-lactylated BSA (2.5 mg ml^−1^) were obtained using analogous procedures. To prepare *N*-ε-carboxyethylated BSA, 10 mM sodium pyruvate was added to 0.5 mg of BSA (in 500 µl of water), and the mixture was kept at 37 °C on an Eppendorf thermomixer at 1,000 rpm for 1 h. Then, 40 µl of 0.6 M NaBH_3_CN was added, and the mixture was incubated for another hour. The reaction mixture was centrifuged in a 3 kDa ultrafiltration tube (12,000*g*, 4 min) and washed three times with PBS. *N*-ε-carboxyethylated BSA was recovered and dissolved in PBS to a concentration of 2.5 mg ml^−1^. Chemical modification of BSA by MGO was carried out as follows. One millimolar MGO was added to 0.5 mg BSA (in 500 µl of PBS, pH 8.0), and the pH of the mixture was adjusted to 8–9 by adding 1 M NaOH. The reaction was kept at 37 °C on an Eppendorf thermomixer at 1,000 rpm for 24 h. The reaction mixture was centrifuged in a 3 kDa ultrafiltration tube (12,000*g*, 4 min) and washed three times with PBS. The resulting BSA was dissolved in PBS to a concentration of 2.5 mg ml^−1^. Chemical lactylation and *N*-ε-carboxyethylation of synthetic peptides (or peptide libraries) were accomplished similarly. Chemically modified peptides were desalted with C18 columns, and then used for coelution experiments.

### Preparation of histone peptide standards

#### General methods

All reagents and solvents were of analytical grade and were used without further purification as obtained from commercial suppliers. HPLC–MS was performed on a Phenomenex Kinetex column (1.7 µm, 50 × 2.10 mm) using a Waters Acquity ultra-HPLC system. Gradient A with eluent I (0.1% HCOOH in H_2_O) and eluent II (0.1% HCOOH in MeCN) rising linearly from 0 to 95% of II was applied during *t* = 0‒5.20 min at a flow rate of 0.6 ml min^−1^. Preparative reversed-phase HPLC purification was performed on a C18 Phenomenex Luna column (5 μm, 100 Å, 250 × 20 mm) or a C18(2) Phenomenex Luna column (5 μm, 250 × 21.2 mm) using an Agilent 1260 LC system equipped with a diode array ultraviolet (UV) detector and an evaporative light scattering detector. Gradient B with eluent III (H_2_O/MeCN/trifluoroacetic acid (TFA), 95:5:0.1, v-v:v) and eluent IV (0.1% TFA in MeCN) rising linearly from 0 to 20–25% of eluent IV was applied during *t* = 5–35 min at a flow rate of 20 ml min^−1^. Analytical HPLC of preparative fractions and of the final product were performed on a C18 Infinity Poroshell 120 column (2.7 µm, 100 × 3.0 mm) using an Agilent 1260 Infinity II series system equipped with a diode array UV detector using eluent III and eluent IV, rising linearly from 0 to 50% of eluent IV during *t* = 1–11 min at a flow rate of 1.2 ml min^−1^ at 40 °C. High-resolution MS (HRMS) was performed on a Bruker SolariX ESI (electrospray ionization) instrument.

#### Peptide synthesis

Here, 2-chlorotrityl resin (0.39 g, Iris Biotech, cat. no. BR-1060.0025, loading 1.55 mmol g^−1^) was swollen in CH_2_Cl_2_ (4 ml) for 5 min. Then, the solvent was removed by suction and the resin was incubated with a solution of Fmoc-Lys(Boc)-H or Fmoc-Arg(Pbf)-H (0.38 mmol, 0.63 equiv.) and ^*i*^Pr_2_EtN (0.22 ml, 1.26 mmol, 2.1 equiv.) in CH_2_Cl_2_ (4 ml) for 2 h. Loaded resins were washed with CH_2_Cl_2_–MeOH–^*i*^Pr_2_EtN (17:2:1, 3 × 5 ml), CH_2_Cl_2_ (3 × 5 ml), dimethylformamide (DMF) (2 × 5 ml) and CH_2_Cl_2_ (2 × 5 ml) and resin loading was determined following a standard procedure based on Fmoc group cleavage (Lys resin, 0.40 mmol g^−1^; Arg resin, 0.28 mmol g^−1^). Then, standard Fmoc/^*t*^Bu solid-phase peptide synthesis was performed on a Biotage SyroWave automated synthesizer with protected amino acids; Fmoc-Ala-OH, Fmoc-Glu(^*t*^Bu)-OH, Fmoc-Gly-OH, Fmoc-Leu-OH, Fmoc-Lys(Alloc)-OH, Fmoc-Pro-OH, Fmoc-Ser(^*t*^Bu)-OH and Fmoc-Thr(^*t*^Bu)-OH, and Boc-Gly-OH, Boc-Gln(Trt)-OH and Boc-Pro-OH for the final coupling. The syntheses were performed on a 50 µmol scale and Fmoc deprotection steps were performed twice with piperidine–DMF (first 2:3 (v:v), 3 min; then 1:4 (v:v), 12 min), followed by washing of the resin with DMF (5 × 45 s). Coupling reactions were performed with Fmoc-Xaa-OH in DMF (400 µl, 0.4 M, 4.0 equiv.), HBTU in DMF (420 µl, 0.38 M, 4.0 equiv.) and ^*i*^Pr_2_NEt in NMP (200 µl, 1.6 M, 8.0 equiv.) for 40 min, followed by washing with DMF (3 × 45 s).

On-resin Alloc deprotection was achieved by addition of borane dimethylamine complex (14.7 mg, 0.250 mmol, 5.0 equiv.) and Pd(PPh_3_)_4_ (5.8 mg, 0.005 mmol, 0.1 equiv.) in anhydrous CH_2_Cl_2_ (2.5 ml) to the resin (2 × 15 min, room temperature, with 2 × 4 ml of CH_2_Cl_2_ wash in between deprotections). Resins were then washed with CH_2_Cl_2_ (3 × 4 ml), DMF (3 × 4 ml) and CH_2_Cl_2_ (3 × 4 ml).

Formation of the K_ce_ side chain modification was performed by incubation of the resin with 2-bromopropionic *tert*-butyl ester (41.5 µl, 0.250 mmol, 5.0 equiv.) and ^*i*^Pr_2_Net (43.5 µl, 0.250 mmol, 5.0 equiv.) in DMF (2.5 ml) at 65 °C (5 h), followed by washing with DMF (3 × 4 ml) and CH_2_Cl_2_ (3 × 4 ml). Formation of the K_d-la_ modification was performed on a 25 µmol scale by addition of d-lactic acid (11.3 mg, 0.125 mmol, 5.0 equiv.), HATU (46.7 mg, 0.123 mmol, 4.9 equiv.) and ^*i*^Pr_2_NEt (35 µl, 0.200 mmol, 8.0 equiv.) in DMF (1.25 ml) at room temperature (2 × 45 min), followed by washing with DMF (3 × 4 ml) and CH_2_Cl_2_ (3 × 4 ml).

Cleavage and global deprotection was achieved with TFA–H_2_O–^*i*^Pr_3_SiH, (95:2.5:2.5, v-v:v) for 2 h at room temperature, followed by concentration under a stream of N_2_, trituration with ice-cold diethyl ether and centrifugation at 1,600*g* (3 min, room temperature). Supernatants were discarded and pellets were washed twice with ice-cold diethyl ether and dried under a stream of N_2_. Peptides were purified by preparative HPLC, and fractions containing the desired peptide were identified by HPLC–MS, lyophilized and final peptide purity was verified by analytical HPLC.

##### H4K8ce

(H-Gly-Gly-Lys(ce)-Gly-Leu-Gly-Lys-OH): *t*_R_ = 2.60 min, 98% purity, 16% yield. HRMS, *m*/*z* [M+H]^+^ calculated for C_29_H_54_N_9_O_10_^+^ 688.39882, found 688.39953.

##### H3K23ce

(H-Gln-Leu-Ala-Thr-Lys(ce)-Ala-Ala-Arg-OH): *t*_R_ = 3.33 min, 93% purity, 2% yield. HRMS, *m*/*z* [M+H]^+^ calculated for C_39_H_72_N_13_O_13_^+^ 930.53671, found 930.53783.

##### H2BK5ce

(H-Pro-Glu-Leu-Ala-Lys(ce)-Ser-Ala-Pro-Ala-Pro-Lys-OH): *t*_R_ = 4.04 min, 99% purity, 31% yield. HRMS, *m*/*z* [M+H]^+^ calculated for C_53_H_90_N_13_O_17_^+^ 1180.65721, found 1180.65624.

##### H4K8(d-la)

(H-Gly-Gly-Lys(d-la)-Gly-Leu-Gly-Lys-OH): *t*_R_ = 3.34 min, 98% purity, 7% yield. HRMS, *m*/*z* [M+H]^+^ calculated for C_29_H_54_N_9_O_10_^+^ 688.39882, found 688.39904.

##### H3K23(d-la)

(H-Gln-Leu-Ala-Thr-Lys(d-la)-Ala-Ala-Arg-OH): *t*_R_ = 3.79 min, 94% purity, 20% yield. HRMS, *m*/*z* [M+H]^+^ calculated for C_39_H_72_N_13_O_13_^+^ 930.53671, found 930.53689.

##### H2BK5(d-la)

(H-Pro-Glu-Leu-Ala-Lys(d-la)-Ser-Ala-Pro-Ala-Pro-Lys-OH): *t*_R_ = 4.39 min, 99% purity, 20% yield. HRMS, *m*/*z* [M+H]^+^ calculated for C_53_H_90_N_13_O_17_^+^ 1180.65721, found 1180.65721.

The HPLC traces of these peptides are shown in Supplementary Information.

### Cells and cell culture

MCF-7, HEK293T and HepG2 cells were sourced from the American Type Culture Collection and were maintained in DMEM, which was supplemented with 10% fetal bovine serum (FBS) and 1% GlutaMAX (Gibco). To generate GLO1-, GLO2- and GLO1/2-deficient HEK293T cell lines, cells were transfected with the pSpCas9(BB)-2A-GFP vector (Addgene no. 48138) expressing Cas9 and single-guide RNAs targeting exon 2 of GLO1 (AGGATCCTTCACTCGTAGCATGG) and exon 4 of GLO2 (TACGGGGGTGACGACCGTATCGG). Single-cell-derived clonal lines were screened and confirmed for GLO1 and GLO2 deficiency by sequencing with primers TTCCTAGTTAAGGCGGCACAGG (for GLO1) and ATGAAGGTAGAGGTGCTGCCTGC (for GLO2) and western blot analysis. The LDH-deficient HepG2 cell line was reported previously^11^.

### Stable isotope tracing

Human HepG2 cells were cultured in complete media (DMEM, high glucose, supplemented with 10% FBS and 1% GlutaMAX). Before labeling by ^13^C_3_ sodium l-lactate, cells were washed once with warm PBS and replenished with fresh complete media. ^13^C_3_ sodium l-lactate (25 mM) was added into the media and cells were collected at indicated time points. To be labeled by U-^13^C_6_ d-glucose, cells were washed once with warm PBS and replenished with fresh media lacking glucose (DMEM, no glucose, supplemented with 10% FBS and 1% GlutaMAX). U-^13^C_6_ d-glucose (25 mM) was added into the media and cells were collected at indicated time points.

### Immunoblotting

For western blotting, whole-cell lysates (20 µg) were separated by 15% SDS–PAGE and then transferred to polyvinylidene fluoride membranes. For dot blotting, 1–1,000 ng of sample was spotted onto the nitrocellulose membrane and allowed to dry. The membranes were first blocked with a 3% solution of BSA in TBST (tris-buffered saline with Tween) (20 mM Tris-HCl, pH 7.6, 150 mM NaCl, 0.1% Tween-20) at room temperature for 1 h. Subsequently, the membranes were incubated with the primary antibodies, which were diluted in a solution containing 1% BSA in TBST, in a cold room environment overnight. After the primary antibody incubation, the membranes were thoroughly washed four times with TBST and then incubated with secondary antibodies (prepared in 1% BSA, TBST) at room temperature for 1 h. The membranes were then developed using a Konica film processor (SRX-101A).

### HPLC‒MS/MS analysis of histone peptides

#### Histone extraction

Histones from cultured human MCF-7 cells were extracted using a previously published method with minor modifications^37^. Briefly, cell pellets from MCF-7 were suspended in cold extraction buffer (10 mM HEPES, pH 8.0, 0.34 M sucrose, 10 mM KCl, 1.5 mM MgCl_2_, 0.1% Triton X-100) and incubated on ice for 30 min. After centrifugation (2,000*g*, 10 min), the resulting pellets were carefully resuspended in a no-salt buffer (3 mM EDTA and 0.2 mM EGTA) and incubated on ice for 30 min. Following this incubation, the samples were centrifuged again (6,500*g*, 5 min), and the resulting pellets were resuspended in a solution of 0.4 N H_2_SO_4_ and transferred to a cold room to incubate overnight. The supernatants were precipitated by adding 20% (v/v) trichloroacetic acid. The resulting precipitates contained histones and were washed twice with cold acetone and dried at 4 °C.

#### Peptide enrichment

Extracted histones were first digested into peptides by trypsin. The resulting peptides were incubated with nProtein A Sepharose beads (Cytiva) preconjugated with pan anti-K_l-la_, pan anti-K_d-la_ or pan anti-K_l-la_ antibodies, or their mixture, in a cold room overnight. The beads were washed three times with NETN buffer (50 mM Tris-HCl, pH 8.0, 100 mM NaCl, 1 mM EDTA, 0.5% NP-40), twice with ETN buffer (50 mM Tris-HCl, pH 8.0, 100 mM NaCl, 1 mM EDTA) and once with purified water to remove impurities. Peptides were eluted using 0.1% TFA and dried using a SpeedVac system (Thermo Fisher Scientific).

#### HPLC‒MS/MS conditions

Peptide samples were injected onto a custom-made C18-packed capillary column (12 cm length by 75 μm inner diameter, Dr. Maisch GmbH), and analyzed using an Orbitrap Exploris 480 mass spectrometer coupled with an EASY-nLC 1000 system (Thermo Fisher Scientific). The HPLC used a gradient of 5–35% buffer B (0.1% formic acid in acetonitrile) in buffer A (0.1% formic acid in water) over 20 min with a flow rate of 0.3 μl min^−1^. In positive-ion mode, full-scan mass spectra were acquired from *m*/*z* 300 to 1,400 at a resolution of 60,000. Data-dependent MS/MS was performed on the 15 most intense ions at a resolution of 15,000 using higher-energy collisional dissociation with parameters set for an isolation window of 2.0 *m*/*z*, charge 2+, collision energy 30% and dynamic exclusion after two occasions within 20 s.

#### Database searching

Data were collected by Xcalibur installed on the mass spectrometer (Thermo Fisher Scientific), and MS/MS spectra were queried against the reverse, concatenated UniProt human FASTA database using ProLuCID^38^. For histone samples, no modification was set to search ‘light’ peptides and a static modification for heavy lysine incorporation (+8.0142) was set to search ‘heavy’ peptides. The maximum missed cleavage site number was 2. For immunoprecipitation samples, the database search was performed using the CHiMA 2.0 strategy^39^. Specifically, four variable modifications (+72.02113 Da for K_l-la_/K_d-la_/K_ce_, +42.01056 Da for K_ac_, +14.01565 Da for lysine and arginine mono-methylation) were used to search ‘light’ peptides. An additional static modification for heavy lysine incorporation (+8.0142) was set to search ‘heavy’ peptides. The maximum missed cleavage site number was 4. The precursor and fragmentation tolerances were 10 and 40 ppm, respectively, for all searches. ProLuCID search results were filtered and assembled by DTASelect v.2.0 (ref. ^40^) with a criterion of at least 1% FDR or more than 50% b/y ion coverage, for histone data searches and immunoprecipitation data searches. Quantification of heavy/light ratios was performed by CIMAGE^41^. The peptide-spectrum matches for all quantified K_l-la_ peptides were manually verified.

### Chiral derivatization and LC‒MS analysis of amino acids

#### Chiral derivatization assay

Immunoprecipitated peptides were digested into amino acids by leucine aminopeptidase (3.6 µl, 0.5 U) in 100 µl of buffer (5 mM MgCl_2_, 100 mM NH_4_HCO_3_, pH 7.9) at 37 °C for 24 h. The mixture was filtered using a 3 kDa centrifugal filter (MilliporeSigma) to collect free amino acids in the eluted solution, which was evaporated to dryness in a vacuum centrifuge (Thermo Fisher Scientific). For chemical derivatization of lactyl lysine standards or digested samples, 1 µg of standards or dried samples was first reconstituted in 100 µl of anhydrous solution containing acetonitrile:pyridine (9:1, v:v). (*R*)-(−)-MTPA-Cl (1 µl), the derivatization reagent, was added to the solution and the reaction mixture was gently vortexed for 2 h at room temperature. After the reaction, the samples were centrifuged for 10 min at 14,000*g*. The supernatant was desalted with C18 Zip-tips (Waters Corporation), evaporated to dryness in a vacuum centrifuge and stored at −80 °C before analysis. Heavy lysine-labeled samples were spiked with nonisotopic d- and l-lactyl lysine standards, and then analyzed in a Fusion Lumos Tribrid mass spectrometer equipped with a Dionex Ultimate 3000 nanoLC system (Thermo Fisher Scientific).

#### HPLC–MS/MS conditions

A 15 cm long, 75 μm inner diameter in-house-packed Bridged Ethylene Hybrid C18 (1.7 μm, 130 Å, Waters Corporation) column was used for HPLC separation. The mobile phases used in the experiments were buffer A (0.1% formic acid in water) and buffer B (0.1% formic acid in water acetonitrile). The HPLC gradient was set as follows: 0–18.33 min, 3% buffer B; 18.33–28 min 3–40% buffer B; 28–58 min, 40–64% buffer B; 58–63 min, 64–95% buffer B; 63–83 min, 95% buffer B and 83–93 min, 95–3% buffer B. The flow rate was 300 nl min^−1^. MS was operated in the parallel reaction monitoring mode using negative electrospray ionization mode for data acquisition. MS1 spectra were collected over the *m*/*z* range of 300–800 at a mass resolution of 120,000. Targeted MS2 spectra were collected over the *m*/*z* range of 60–800 at a mass resolution of 15,000, triggered by a scheduled inclusion list. Fragmentation was conducted by higher-energy collisional dissociation with collision energy value set at 20% normalized collision energy after optimization. Both MS1 and MS2 spectra were collected using the Orbitrap with a maximum automatic gain control value of 5.0 × 10^5^.

### Lactyl-CoA analysis and LC–MS quantification

Lactyl-CoA was quantified by LC–MS/MS as previously described^15^. Briefly, HepG2 cell pellets were collected in 1 ml of 10% (w/v) trichloroacetic acid at 4 °C, then frozen and shipped for analysis. Fifty microliters of ^13^C_3_^15^N_1_ acyl-CoA mix were added as an internal standard, which was prepared as previously described from ^13^C_3_^15^N_1_ pantothenate in yeast culture^42^. Samples were mixed, sonicated and centrifuged at 17,000*g* at 4 °C for 10 min. To extract metabolites from the resulting supernatant, Oasis HLB solid phase extraction cartridges (Waters Corporation) were washed with 1 ml of methanol and equilibrated with 1 ml of water. The sample was loaded and desalted with 1 ml of water and eluted with 1 ml of eluting buffer (25 mM ammonium acetate in methanol). The eluent was evaporated to dryness under nitrogen gas, and then resolubilized in 50 µl of 5% (w/v) 5-sulfosalicylic acid in water. Ten microliters of the sample were injected into an Ultimate 3000 UHPLC system with an HSS T3 column (2.1 × 150 mm, 3.5 µm Waters Corporation) and connected to a Q Exactive Plus mass spectrometer (Thermo Fisher Scientific). The analysis used a flow rate of 0.2 ml min^−1^ and a gradient elution starting with 100% buffer A (5 mM ammonium acetate in water) for 3 min, then 20% buffer B (5 mM ammonium acetate in acetonitrile:water, 95:5) for 2 min, followed by 100% B for 7 min. After washing with buffer C (acetonitrile:water:formic acid, 80:20:0.1) at 0.275 ml min^−1^ for 3.5 min, the system re-equilibrated with 100% A for 5 min. Lactyl-CoA was analyzed in single-ion monitoring or data-independent acquisition mode using the [M+H]^+^ ion in full scan or the [M-507+H]^+^ product ion with a 5 ppm window with TraceFinder v.4.1 (Thermo Fisher Scientific). Absolute amounts were calculated from linear calibration curves that were created by serial dilution of synthetically prepared lactyl-CoA. Sample preparation and analysis of calibration were identical to the unknown samples. Analysts were blinded to sample identity during sample preparation and analysis.

### Graphing and statistical analyses

GraphPad Prism v.9 software was used for graphing and statistical analysis. Data are shown as mean ± standard error of the mean (s.e.m.). For comparison between two groups, datasets were analyzed by a two-tailed Student’s *t*-test. For data involving two independent variables, we employed a two-way analysis of variance, followed by Tukey’s test for multiple comparisons.

### Reporting summary

Further information on research design is available in the Nature Portfolio Reporting Summary linked to this article.

## Online content

Any methods, additional references, Nature Portfolio reporting summaries, source data, extended data, supplementary information, acknowledgements, peer review information; details of author contributions and competing interests; and statements of data and code availability are available at 10.1038/s41589-024-01680-8.

## Supplementary information

Supplementary Information Supplementary Figs. 1–6.

Reporting Summary

## Source data

Source Data Fig. 5 Unmodified blots.

Source Data Fig. 6 Unmodified blots.

Source Data Fig. 6 Statistical source data.

Source Data Extended Data Fig. 8 Unmodified blots.

## Data Availability

All data needed to evaluate the conclusions in the paper are present in the paper and/or Supplementary Information. The raw MS proteomics data have been deposited to the iProX database with the dataset identifier IPX0006076001. Source data are provided with this paper.
