# Supplementary material for: Lysine l-lactylation is the dominant lactylation isomer induced by glycolysis
Source: Nat Chem Biol. 2024 Jul 19;21(1):91–9. doi: 10.1038/s41589-024-01680-8 (PMC11666458; doi:10.1038/s41589-024-01680-8)
Supplement: Supplementary file 1 — Supplementary Figs. 1–6. [file 41589_2024_1680_MOESM1_ESM.pdf]

nature chemical biology

Supplementary information https://doi.org/10.1038/s41589-024-01680-8

# Lysine L-lactylation is the dominant lactylation isomer induced by glycolysis

In the format provided by the authors and unedited

Supplementary Information includes Supplementary Figs. 1-6.

**Supplementary Fig. 1**

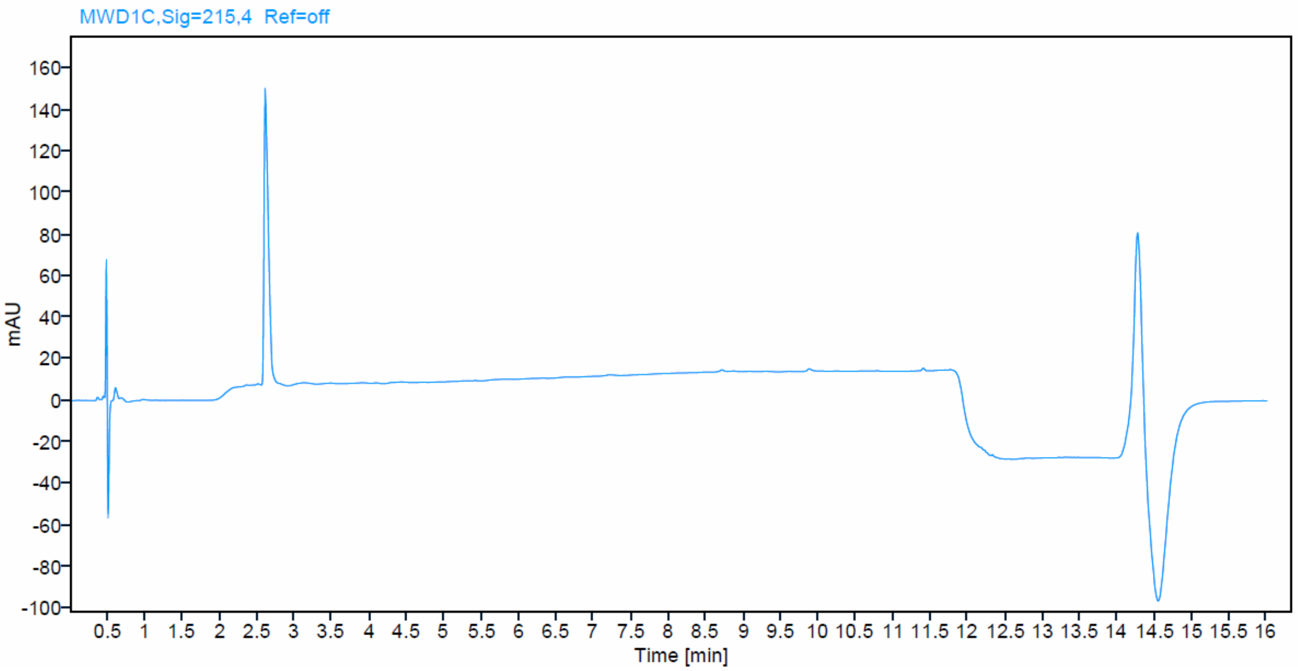


Supplementary Fig. 1. HPLC trace of the synthetic peptide H4K8ce (GGK$_{ce}$GLGK)

## Supplementary Fig. 2

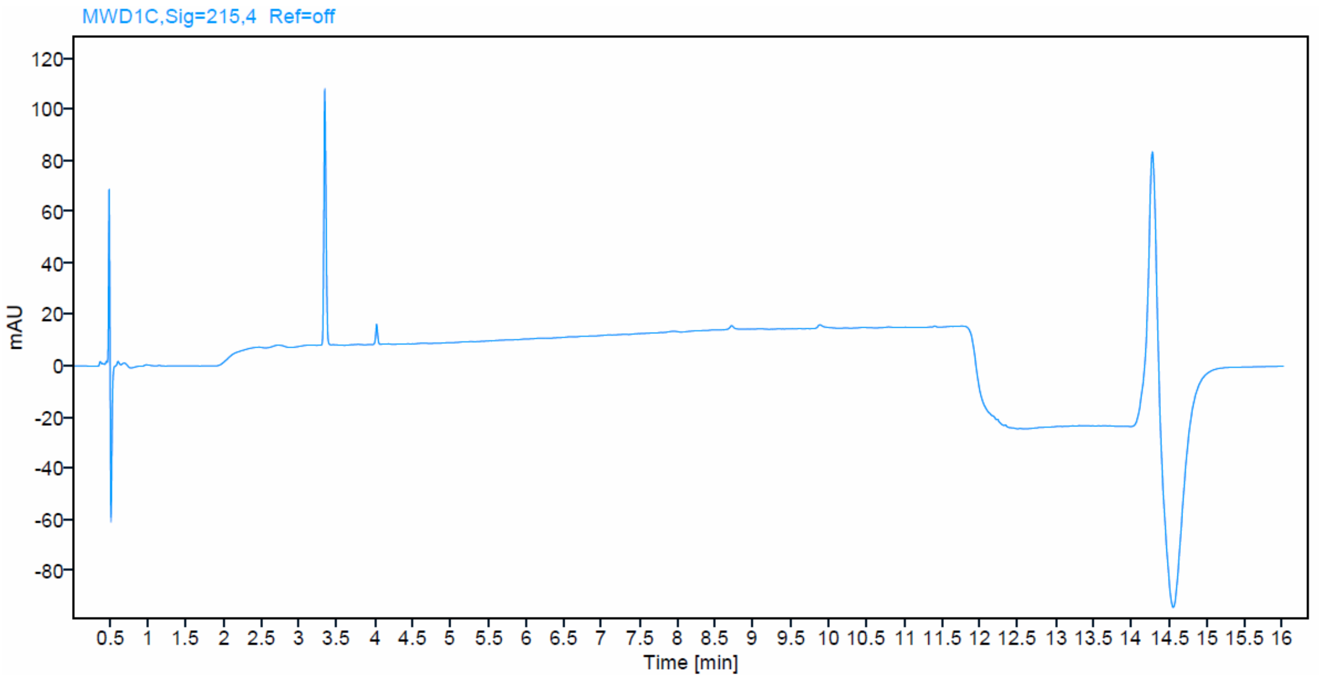


Supplementary Fig. 2. HPLC trace of the synthetic peptide H3K23ce (QLATK$_{ce}$AAR)

**Supplementary Fig. 3**

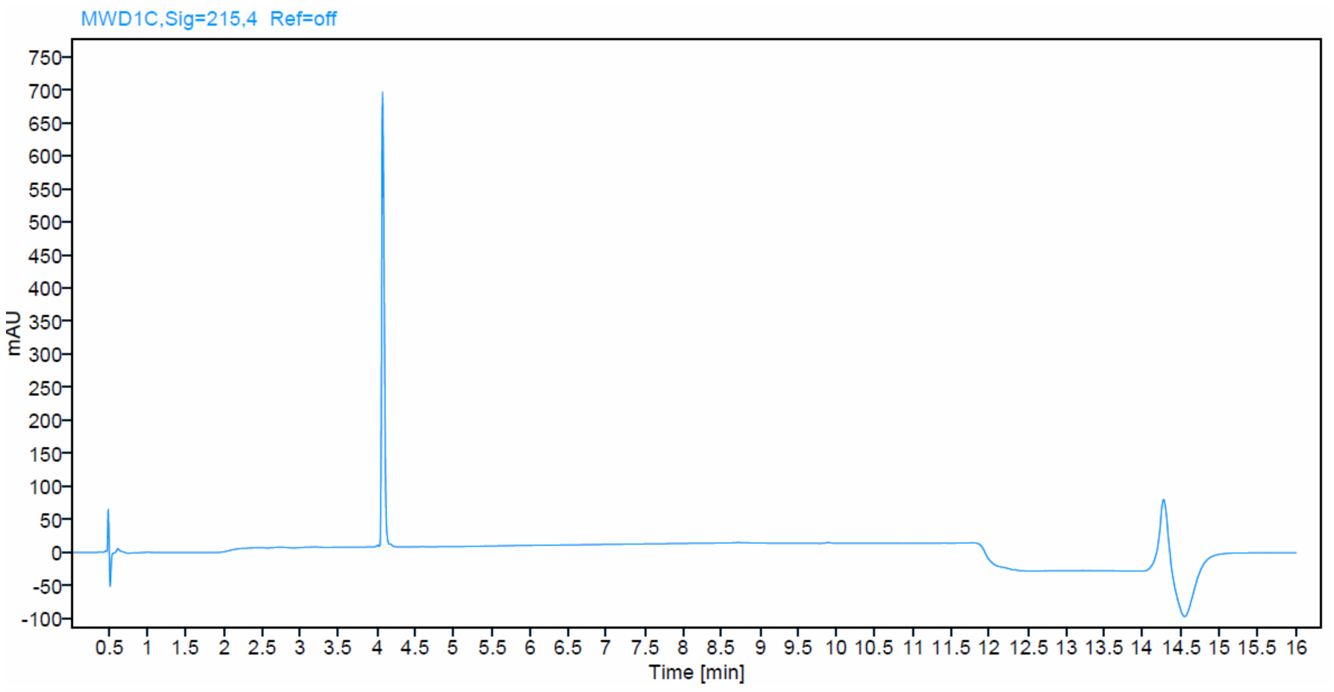


Supplementary Fig. 3. HPLC trace of the synthetic peptide H2BK5ce (PELAK$_{ce}$SAPAPK)

**Supplementary Fig. 4**

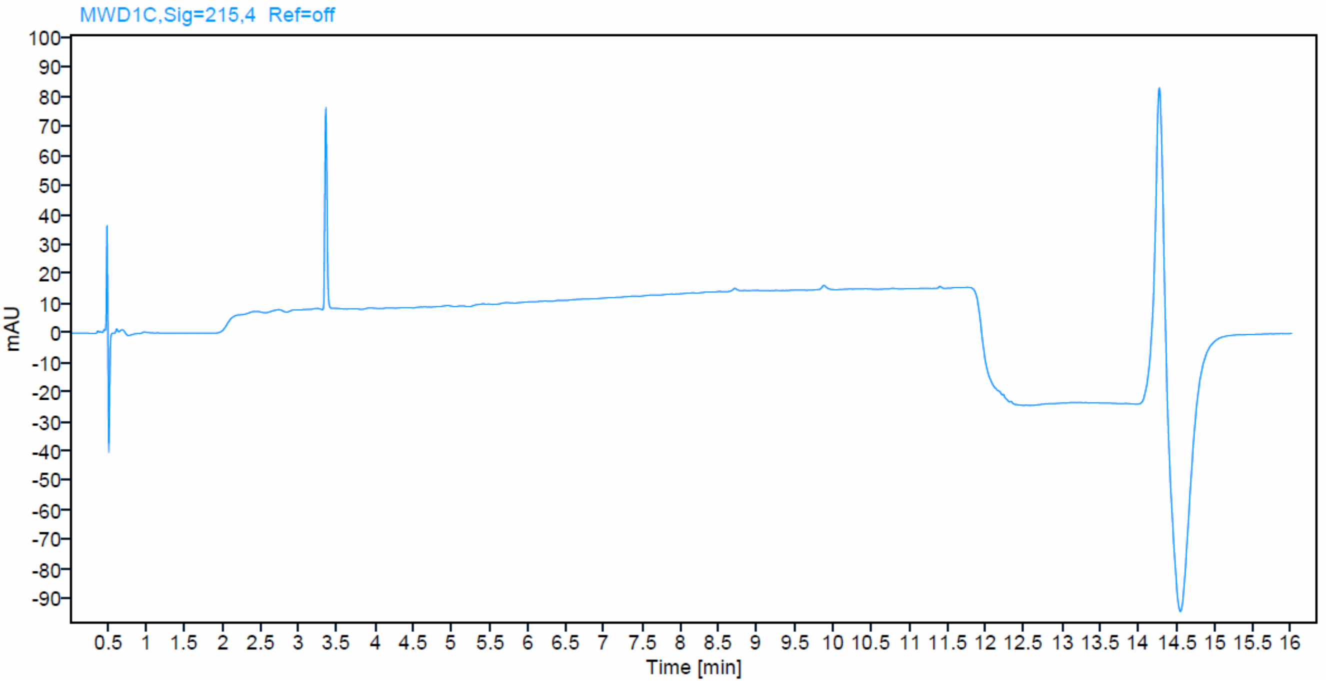


Supplementary Fig. 4. HPLC trace of the synthetic peptide H4K8$_{D\text{-}la}$ (GGK$_{D\text{-}la}$GLGK)

**Supplementary Fig. 5**

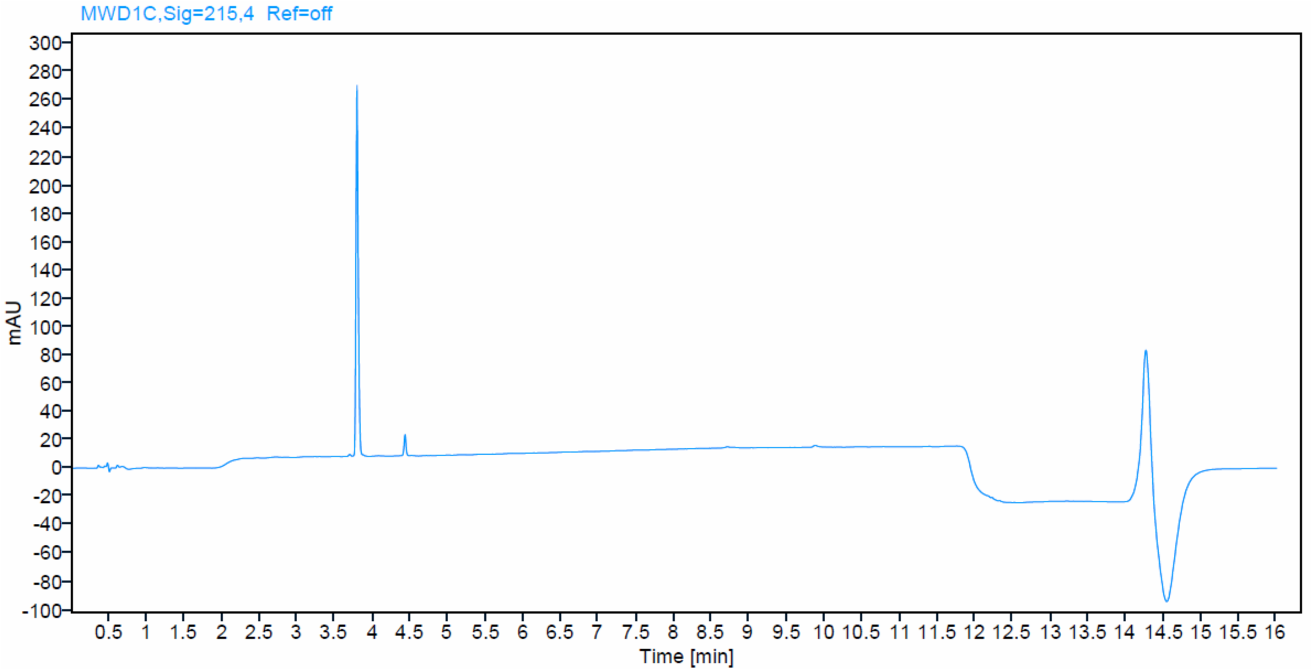


Supplementary Fig. 5. HPLC trace of the synthetic peptide H3K23(D-la) (QLATK$_{\text{D-la}}$AAR)

**Supplementary Fig. 6**

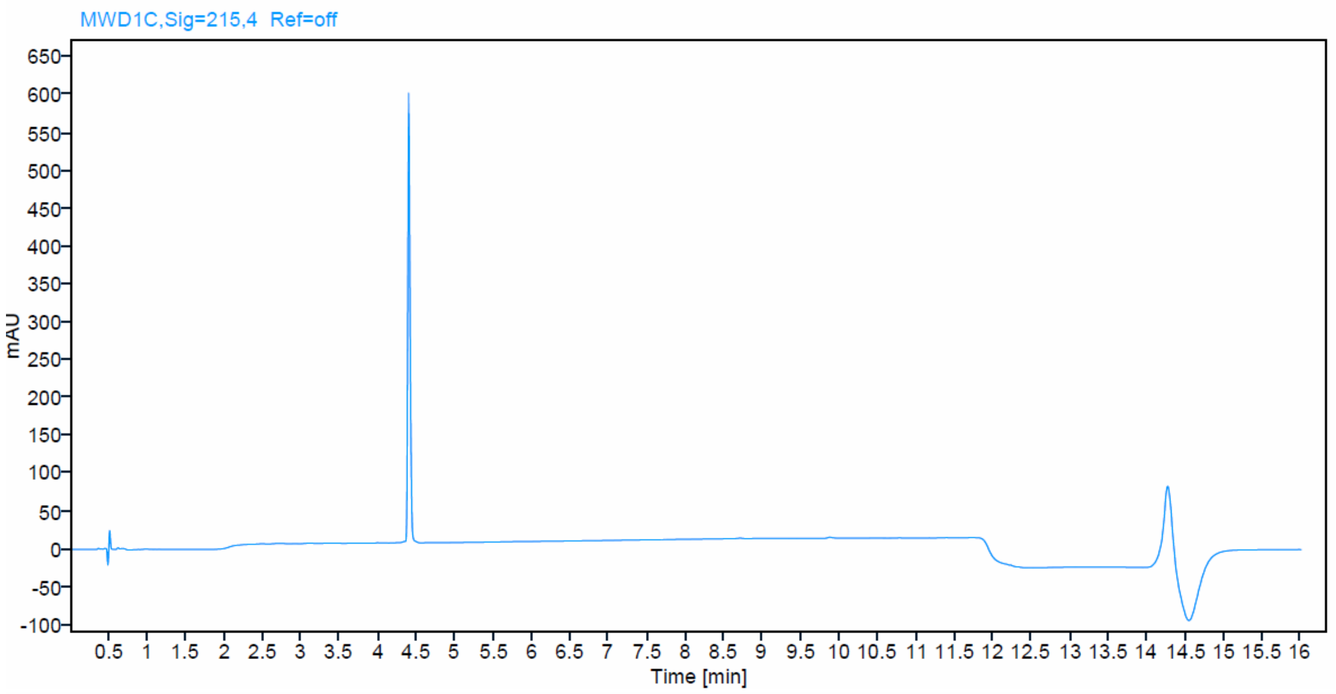


Supplementary Fig. 6. HPLC trace of the synthetic peptide H2BK5(D-la) (PELAK$_{D\text{-}la}$SAPAPK)
