# Supplementary material for: Lysine l-lactylation is the dominant lactylation isomer induced by glycolysis
Source: Nat Chem Biol. 2024 Jul 19;21(1):91–9. doi: 10.1038/s41589-024-01680-8 (PMC11666458; doi:10.1038/s41589-024-01680-8)
Supplement: Supplementary file 2 — Reporting Summary [file 41589_2024_1680_MOESM2_ESM.pdf]

nature portfolio

Corresponding author(s): Di Zhang, Lunzhi Dai, Lingjun Li, Yingming Zhao

Last updated by author(s): May 30, 2024

Reporting Summary

Nature Portfolio wishes to improve the reproducibility of the work that we publish. This form provides structure for consistency and transparency in reporting. For further information on Nature Portfolio policies, see our Editorial Policies and the Editorial Policy Checklist.

Statistics

For all statistical analyses, confirm that the following items are present in the figure legend, table legend, main text, or Methods section.

n/a	Confirmed	
☐	☒	The exact sample size (n) for each experimental group/condition, given as a discrete number and unit of measurement
☐	☒	A statement on whether measurements were taken from distinct samples or whether the same sample was measured repeatedly
☐	☒	The statistical test(s) used AND whether they are one- or two-sided<br>*Only common tests should be described solely by name; describe more complex techniques in the Methods section.*
☒	☐	A description of all covariates tested
☒	☐	A description of any assumptions or corrections, such as tests of normality and adjustment for multiple comparisons
☐	☒	A full description of the statistical parameters including central tendency (e.g. means) or other basic estimates (e.g. regression coefficient) AND variation (e.g. standard deviation) or associated estimates of uncertainty (e.g. confidence intervals)
☐	☒	For null hypothesis testing, the test statistic (e.g. F, t, r) with confidence intervals, effect sizes, degrees of freedom and P value noted<br>*Give P values as exact values whenever suitable.*
☒	☐	For Bayesian analysis, information on the choice of priors and Markov chain Monte Carlo settings
☒	☐	For hierarchical and complex designs, identification of the appropriate level for tests and full reporting of outcomes
☒	☐	Estimates of effect sizes (e.g. Cohen's d, Pearson's r), indicating how they were calculated

Our web collection on statistics for biologists contains articles on many of the points above.

Software and code

Policy information about availability of computer code

Data collection	XCalibur installed on the mass spectrometer (Thermo Fisher Scientific, Waltham, MA)
Data analysis	ProLuCID, DTASelect 2.0, TraceFinder 4.1, CIMAGE2.0, GraphPad Prism 9

For manuscripts utilizing custom algorithms or software that are central to the research but not yet described in published literature, software must be made available to editors and reviewers. We strongly encourage code deposition in a community repository (e.g. GitHub). See the Nature Portfolio guidelines for submitting code & software for further information.

Data

Policy information about availability of data

All manuscripts must include a data availability statement. This statement should provide the following information, where applicable:

- Accession codes, unique identifiers, or web links for publicly available datasets
- A description of any restrictions on data availability
- For clinical datasets or third party data, please ensure that the statement adheres to our policy

All data needed to evaluate the conclusions in the paper are present in the paper and/or the Supplementary Information. The raw mass spectrometry proteomics data have been deposited to the iProX database with the dataset identifier IPX0006076001.

Research involving human participants, their data, or biological material

Policy information about studies with human participants or human data. See also policy information about sex, gender (identity/presentation), and sexual orientation and race, ethnicity and racism.

Reporting on sex and gender	N/A
Reporting on race, ethnicity, or other socially relevant groupings	N/A
Population characteristics	N/A
Recruitment	N/A
Ethics oversight	N/A

Note that full information on the approval of the study protocol must also be provided in the manuscript.

Field-specific reporting

Please select the one below that is the best fit for your research. If you are not sure, read the appropriate sections before making your selection.

☒ Life sciences ☐ Behavioural & social sciences ☐ Ecological, evolutionary & environmental sciences

For a reference copy of the document with all sections, see nature.com/documents/nr-reporting-summary-flat.pdf

Life sciences study design

All studies must disclose on these points even when the disclosure is negative.

Sample size	No formal sample-size calculations ere performed. Our sample sizes were chosen based on a combination of prior literature, pratical considerations, and preliminary data analyses.
Data exclusions	None.
Replication	All experiments were successfully repeated for at least 3 times.
Randomization	Sample order on LC/MS runs was randomized per run. For all other experiments and measurements, samples were randomly assigned to experimental groups.
Blinding	Data collection and analysis were performed by different Investigators who were blinded to each other.

Reporting for specific materials, systems and methods

We require information from authors about some types of materials, experimental systems and methods used in many studies. Here, indicate whether each material, system or method listed is relevant to your study. If you are not sure if a list item applies to your research, read the appropriate section before selecting a response.

Materials & experimental systems

n/a	Involved in the study	
☐	☒	Antibodies
☐	☒	Eukaryotic cell lines
☒	☐	Palaeontology and archaeology
☒	☐	Animals and other organisms
☒	☐	Clinical data
☒	☐	Dual use research of concern
☒	☐	Plants

Methods

n/a	Involved in the study	
☒	☐	ChIP-seq
☒	☐	Flow cytometry
☒	☐	MRI-based neuroimaging

Antibodies

Antibodies used	Pan anti-KL-la (PTM-1401RM, 1:2000 dilution for WB), pan anti-KD-la (ZC288, 1:1500 dilution for WB) and pan anti-Kce (PTM-1701RM, 1:1000 dilution for WB) antibodies were from PTM Biolabs, Inc (Chicago, IL); anti-β-actin (# 4970, 1:6000 dilution for WB) and anti-histone H3 (# 4499, 1:2000 dilution for WB) were purchased from Cell Signaling Technology, Inc (Danvers, MA); anti-

	GLO1 (A4329, 1:1000 dilution for WB) was purchased from ABclonal (Wuhan, China); anti-GLO2 (12146-RP02, 1:1000 dilution for WB) was purchased from Sino Biological (Beijing, China)
Validation	For pan anti-KL-la, pan anti-KD-la, and pan anti-Kce antibodies, specificity of the antibodies were demonstrated in Figure 2, as demonstrated through their reactivity against human, mouse, and bovine antigens.<br><br>β-Actin (13E5) Rabbit mAb, # 4970, Cell Signaling<br>validated in human samples for western blot<br>Nature 2023- "Glioma synapses recruit mechanisms of adaptive plasticity."<br><br>Histone H3 (D1H2) XP® Rabbit mAb, #4499, Cell Signaling<br>validated in human and mouse samples for western blot<br>JCI insight 2023-"High-fat diet plus HNF1A variant promotes polyps by activating β-catenin in early-onset colorectal cancer."<br><br>GLO1 Rabbit mAb (A4329), ABclonal<br>validated in human and mouse samples for western blot. https://abclonal.com.cn/catalog/A4329<br>We have independently confirmed the absence of the GLO1 protein signal in knockout cells through western blot analysis.<br><br>GLO2 Rabbit Polyclonal Antibody, Cat: 12146-RP02, Sino Biological<br>validated in human samples for western blot. https://www.sinobiological.com/antibodies/human-glyoxalase-ii-hagh-12146-rp02<br>We have independently confirmed the absence of the GLO2 protein signal in knockout cells through western blot analysis.

Eukaryotic cell lines

Policy information about cell lines and Sex and Gender in Research

Cell line source(s)	MCF-7, HEK293T, and HepG2 cells were obtained from the American Type Culture Collection
Authentication	None
Mycoplasma contamination	Not tested
Commonly misidentified lines<br>(See ICLAC register)	No commonly misidentified cell lines were used.

Plants

Seed stocks	N/A
Novel plant genotypes	N/A
Authentication	N/A
