# Supplementary material for: Lysine l-lactylation is the dominant lactylation isomer induced by glycolysis
Source: Nat Chem Biol. 2024 Jul 19;21(1):91–9. doi: 10.1038/s41589-024-01680-8 (PMC11666458; doi:10.1038/s41589-024-01680-8)
Supplement: Supplementary file 4 — Unmodified blots. [file 41589_2024_1680_MOESM4_ESM.pdf]

Source Data Figure 6

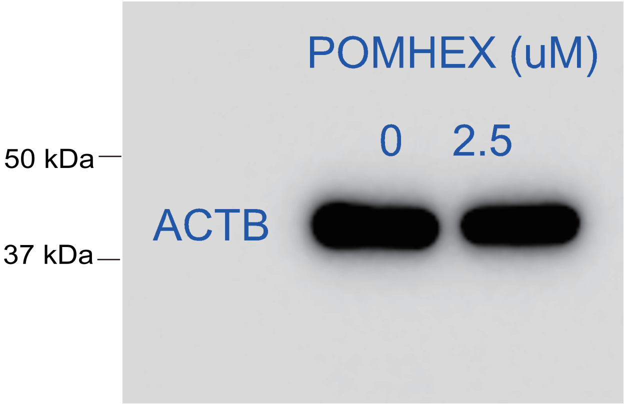

Anti-β-actin

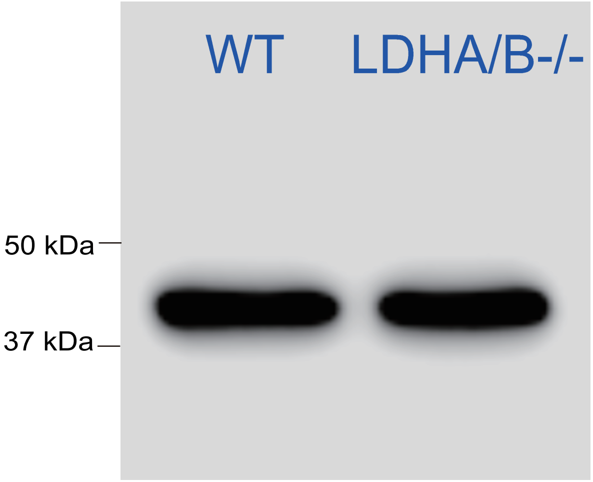

Anti-β-actin

Raw data for Western blots in Figure 6. Uncropped images of western blots displayed in Fig 6e-f.
