# Supplementary material for: Lysine l-lactylation is the dominant lactylation isomer induced by glycolysis
Source: Nat Chem Biol. 2024 Jul 19;21(1):91–9. doi: 10.1038/s41589-024-01680-8 (PMC11666458; doi:10.1038/s41589-024-01680-8)
Supplement: Supplementary file 6 — Unmodified blots. [file 41589_2024_1680_MOESM6_ESM.pdf]

Source Data Extended Data Figure 8

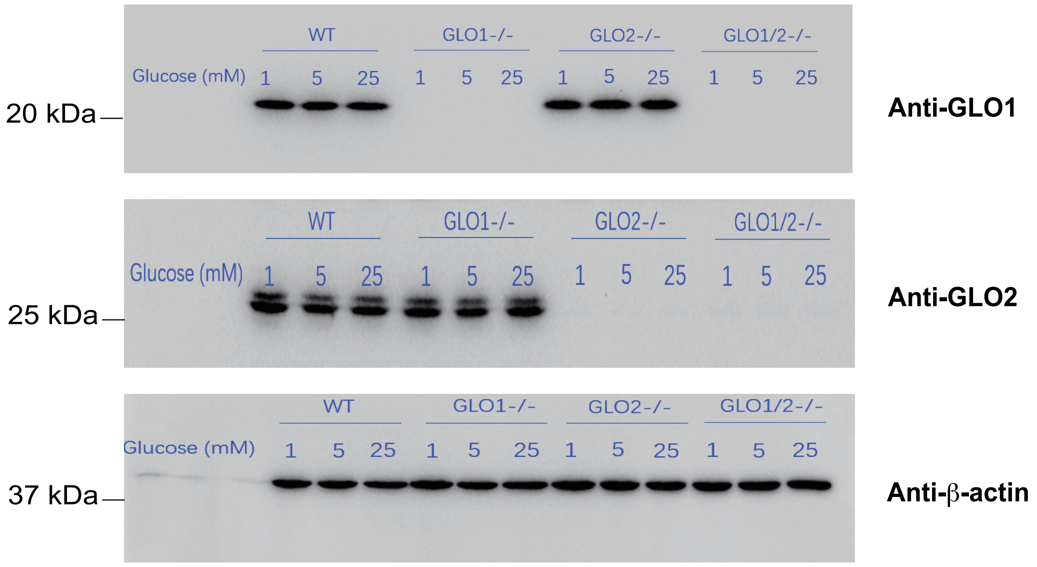

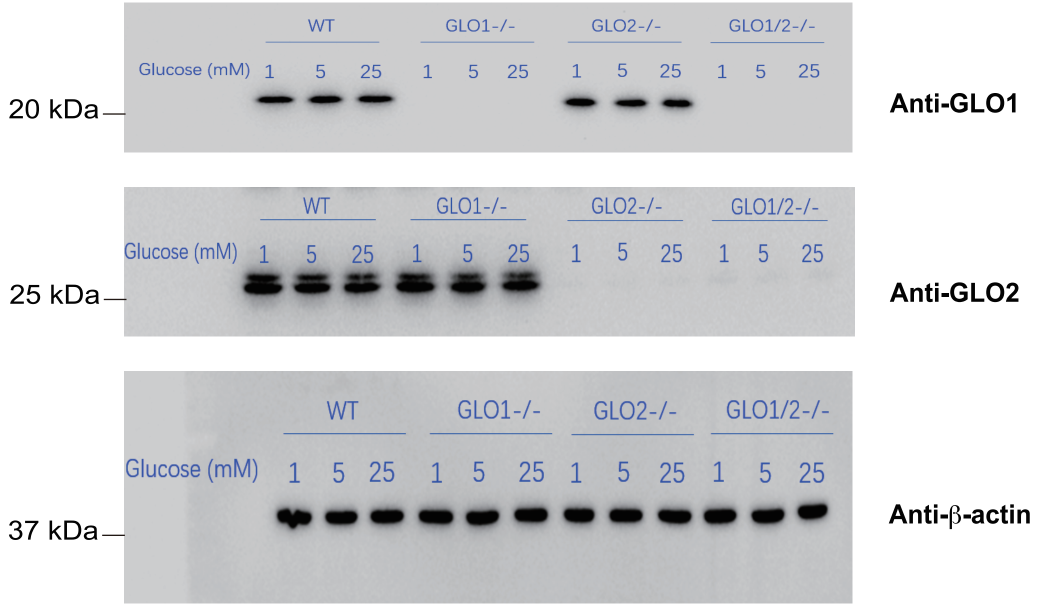

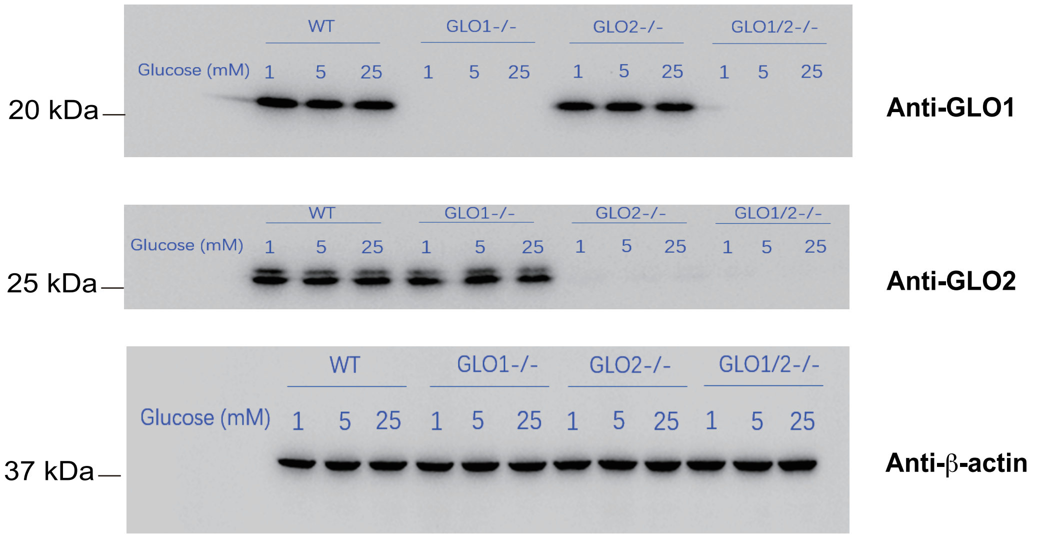

Raw data for Western blots in Extended Data Figure 8. Uncropped images of western blots displayed in Extended Data 8b-d.
